# Long noncoding RNA *BFAL1* mediates enterotoxigenic *Bacteroides fragilis*-related carcinogenesis in colorectal cancer via the RHEB/mTOR pathway

**DOI:** 10.1038/s41419-019-1925-2

**Published:** 2019-09-12

**Authors:** Yujie Bao, Jiayin Tang, Yun Qian, Tiantian Sun, Huimin Chen, Zhaofei Chen, Danfeng Sun, Ming Zhong, Haoyan Chen, Jie Hong, Yingxuan Chen, Jing-Yuan Fang

**Affiliations:** 1 State Key Laboratory for Oncogenes and Related Genes; Key Laboratory of Gastroenterology & Hepatology, Ministry of Health; Division of Gastroenterology and Hepatology; Shanghai Institute of Digestive Disease; Renji Hospital, School of Medicine, Shanghai Jiao Tong University, 145 Middle Shandong Road, 200001 Shanghai, China; 2 Department of Infectious Disease, Shanghai Ninth People’s Hospital, Shanghai Jiao Tong University School of Medicine, 639 Zhizhaoju Road, 200001 Shanghai, China; 3 Department of Gastrointestinal Surgery, Renji Hospital, School of Medicine, Shanghai Jiao Tong University, 200001 Shanghai, China

**Keywords:** Cancer, Cancer epidemiology, Colorectal cancer

## Abstract

Long noncoding RNAs (lncRNAs) contribute to many steps in carcinogenesis and often serve as biomarkers or therapeutic targets for tumor diagnosis and therapy. Although the role of lncRNAs in tumor formation is becoming clear, whether lncRNAs mediate gut microbiota-induced colorectal cancer (CRC) is largely unknown. Enterotoxigenic *Bacteroides fragilis* (ETBF) is a well-known tumor-inducing bacterium in the human gut; however, its tumorigenic effect remains to be explored. In the present study, we revealed the mechanism by which a lncRNA participates in gut bacteria-induced carcinogenesis: *Bacteroides fragilis*-associated lncRNA1 (*BFAL1*) in CRC tissues mediates ETBF carcinogenesis. *BFAL1* was highly expressed in CRC tissues compared with that in adjacent normal tissues. In vitro, *BFAL1* was upregulated in ETBF-treated CRC cells. Mechanistically, ETBF promoted tumor growth via *BFAL1* by activating the Ras homolog, which is the MTORC1 binding/mammalian target of the rapamycin (RHEB/mTOR) pathway. Furthermore, *BFAL1* regulated *RHEB* expression by competitively sponging microRNAs miR-155-5p and miR-200a-3p. Clinically, both high expression of *BFAL1* and high abundance of ETBF in CRC tissues predicted poor outcomes for patients with CRC. Thus, *BFAL1* is a mediator of ETBF-induced carcinogenesis and may be a potential therapeutic target for ETBF-induced CRC.

## Introduction

CRC is one of the most common malignant tumors worldwide, occurring in 5% of the adult population in the United States. Around 250,000 new cases are diagnosed each year, accounting for ~9% of all malignancies in Europe^1–4^. CRC carcinogenesis is controlled by both genetic and environmental factors, in which the gut microbiota plays an important role in CRC formation^5^. Enterotoxigenic *Bacteroides fragilis* (ETBF) is one of the most prevalent species of carcinogenic bacteria in the colon^6^. ETBF is a subtype strain of *Bacteroides fragilis*, which possesses *a bft* gene, encoding *Bacteroides Fragilis* Toxin (BFT); the nontoxigenic *Bacteroides fragilis* (NTBF) subtype lacks the toxin gene^7,8^. Previous studies revealed that BFT targets the epithelial cell tight junctions, resulting in E-cadherin cleavage, enhanced barrier permeability, and Wnt/β-catenin and nuclear factor kappa B (NF-κB) signaling^9^. A recent study showed that BFT promoted the normal-polyp-cancer process^10^. These mechanisms involved genetic mutations in various genes, such as *ICAM1* (intercellular adhesion molecule 1), *AR* (androgen receptor), *JNK* (JUN N-terminal kinase), *MAPK* (mitogen-activated protein kinase), and *NF-κB*^11–13^.

Less than 2% of the human genome comprises protein-coding genes and the vast majority of transcripts consist of noncoding RNAs, representing a shift in our understanding of genome regulation that has emerged recently^14^. It is now apparent that the majority of cellular transcripts do not encode proteins, and many of these transcripts are long noncoding RNAs (lncRNAs)^15^. LncRNAs are transcribed mainly by RNA polymerase II, and are 5ʹ-capped and polyadenylated like most RNAs, yet this class of transcripts has limited coding potential^16^. LncRNAs are involved in numerous biological functions and pathological processes, including development, proliferation, apoptosis, survival, differentiation, and carcinogenesis^17,18^, contributing to gene regulation by different mechanisms^19^. Among the reported mechanisms, some lncRNAs act post-transcriptionally as regulators of splicing, mRNA decay, protein translation, protein stability, or as molecular decoys for microRNAs^20^.

As omics-based technologies have matured, increasing evidence points to the microbial generation of bioactive compounds that affect the transcriptional machinery of host cells^21^. Recent studies have provided insights into the crosstalk between the gut microbiota and the host epigenome, including DNA methylation, histone modification, and noncoding RNAs^22,23^. Commensal microbiota-regulated lncRNAs have been identified in mouse models. Compared with germ-free mice, those that were colonized with specific bacteria displayed a significantly different lncRNA profile, with most of the lncRNAs being transcribed from introns. These lncRNAs contributed to the responses of intestinal epithelial cells to the bacteria^24^. However, these data were solely based upon the bioinformatic data. Therefore, we decided to identify an ETBF-associated lncRNA and explore its molecular mechanism in human CRC carcinogenesis to provide a potential therapeutic target for ETBF-induced CRC.

In the present study, *Bacteroides fragilis*-associated lncRNA1 (*BFAL1*) was identified. The expression profile of *BFAL1* was validated and its function in ETBF-related carcinogenesis was investigated. *BFAL1* mediates ETBF-induced tumor growth by activating the Ras homolog, which is the MTORC1 binding/mammalian target of the rapamycin (RHEB/mTOR) pathway. Further study showed that *BFAL1* competitively bound to miRNAs miR-155-5p and miR-200a-3p to upregulate *RHEB* expression. Clinicopathological information from 96 patients with CRC suggested that *BFAL1* was an independent indicator of prognosis. Thus, the present study might identify a new field of research into how noncoding RNAs respond to microbial signaling and promote CRC carcinogenesis.

## Materials and methods

### CRC tissue specimens

The use of human tissues was performed in accordance with the Declaration of Helsinki and was approved by the ethics committee of Renji Hospital. Written informed consent was obtained from all participants in this study. Cohort 1 represented adult patients with CRC who underwent primary colorectal surgical resections at Renji Hospital and were enrolled from January 2010 to April 2014. All patients were diagnosed as colorectal adenocarcinomas. None of these patients had received radiotherapy or chemotherapy before surgery. Paired tissues (tumors and adjacent normal tissues) were collected and preserved in liquid nitrogen immediately for subsequent study.

### Detection of the amounts of ETBF in paired CRC tissues

To detect the amounts of ETBF in CRC tissues, the total DNA was extracted from the paired CRC tissues by using a QIAamp DNA Mini Kit (QIAGEN, Hilden, Germany). DNA from each specimen was subjected to quantitative real-time PCR (qPCR) to detect the amounts of ETBF. The detected amount of the *bft* gene was normalized to that of the 16 S gene (Supplementary Material Table 1).

### Quantification of mRNAs and microRNAs

The total RNA was isolated from cells by using the TRIzol reagent (Takara, Dalian, China) according to the manufacturer’s protocol. Separation of the nuclear and cytoplasmic fractions was performed by using a PARIS™ Kit (Invitrogen, Carlsbad, CA, USA). To obtain cDNA, 1 µg of RNA was used as a template, and reverse transcription was performed by using a PrimeScript 1st strand cDNA Synthesis Kit (Takara) according to the manufacturer's instructions. Primers for LncRNAs and genes were designed and synthesized by Sangong Biotech, Shanghai, China (Supplementary Material Table 1). For miRNAs, 0.5 µg of the total RNA was reverse transcribed into cDNA by using a specific miRNA stem loop primer. The levels of mRNA and miRNA were assessed quantitatively by using SYBR Green-based qPCR with specifically designed primers (GeneCopoeia, Rockville, MD, USA) (Supplementary Material Table 2). All qPCR reactions were performed by using a 7500 Fast Real-Time PCR System (Applied Biosystems), and all qPCR reagents were purchased from Takara. For each reaction, 1 µL of the RT product was added to 10 µL of 2 × SYBGreen PCR Master Mix. Each sample was analyzed in triplicate. For lncRNAs and mRNAs, *ACTB* (encoding beta actin) was used as an internal normalization control, and for the miRNAs, U6 was used as the internal normalization control. Relative quantification (RQ) was derived from the difference in the cycle threshold (Ct) between the target RNA and internal controls (ΔCt) as compared with control samples (ΔΔCt) by using the equation RQ = 2 –ΔΔCt.

### Cell lines and cell culture

Human CRC cell lines and the human normal colonic epithelial cell line FHC were purchased from American Type Culture Collection (ATCC, Rockefeller, MD, USA). Cells were cultured in a different medium according to the ATCC’s indication (Gibco, Carlsbad, CA, USA). Cells were cultured in 5% CO_2_ in a 37 °C incubator.

### Bacteria strains and the growth condition

The ETBF strain (ATCC 43860) and the NTBF strain ATCC 25285^25,26^ were purchased from ATCC. These two subtypes were cultured in the same medium and under anaerobic conditions. The anaerobic conditions were created by using a DG250 device (Don Whiteley Scientific, West Yorkshire, UK) and comprised an atmosphere of 90% N_2_, 5% CO_2_, and 5% H_2_ at 37 °C. The anaerobic bacteria medium was prepared according to the ATCC indication (modified chopped meat medium). All the ETBF and NTBF treatment experiments in this article used the same bacterial concentration: a multiplicity of infection (MOI) of 500.

### Overexpression of BFAL1 in CRC cells

For ectopic expression, the full-length *BFAL1* cDNA was subcloned into the vector pCDNA3.1 and transfected into HCT116 and DLD-1 cell lines by using the FuGENE HD transfection Reagent (Promega, Madison, WI, USA). The vectors were designed and constructed by Gene Pharma Company (Shanghai, China).

### Transfection of small-interfering RNAs and microRNA mimics and inhibitors

The specified *BFAL1* small-interfering RNAs (siRNAs) siRNA1 and siRNA2, and the specified *RHEB* small RNA, control siRNA were designed to knockdown *BFAL1* or *RHEB* expression in cells. MiR-155-5p mimics and inhibitors and miR-200a-3p mimics and inhibitors were transfected to overexpress or knockdown the relevant microRNAs in cells. All these siRNA and miRNA mimics and inhibitors were designed and synthesized by Gene Pharma Company (Supplementary Material Table 3). Transfection was performed by using the DharmaFECT transfection reagent (GE, Boston, MA, USA) according to the manufacturer's instructions.

### Cell proliferation assays and the cell cycle test

To assay cell proliferation, the CCK-8 (Cell Counting Kit-8, Dojindo, Japan) assay was used according to the manufacturer's instructions. Flow-cytometry analysis was conducted by using a BD LSR Fortessa instrument and PI/RNase Staining Buffer (BD Biosciences, Lake Franklin, NJ, USA) according to the manufacturer’s instructions. The percentages of cells in different phases of the cell cycle were analyzed by using Flowjo software.

### Bioinformatic methods

The potential microRNAs targeting *RHEB* were predicted and validated by using several online databases with different bioinformatic algorithms, such as TargetScan and RNAhybrid. We predicted the potential target genes of miR-155-5p and miR-200a-3p mainly based on a combination of 3ʹ UTR binding sites of target genes and the seed regions of miR-155-5p and miR-200a-3p. Furthermore, the minimum free energy (MFE) values of miRNA–lncRNA hybridization were calculated by using RNAhybrid software to evaluate the binding potential between *BFAL1* and miR-155-5p or miR-200a-3p.

### High-throughput sequencing

For RNA sequencing (RNA-seq), libraries were generated from the total RNA by using TruSeq RNA Sample Preparation v2, according to the manufacturer's protocol. Samples were sequenced on the Illumina HiScanSQ platform (Illumina, San Diego, CA, USA). Reads were mapped to the human genome (Hg19) by using TopHat v2.0.6 (Johns-Hopkins University, Baltimore, MD, USA), and mRNA quantification was performed by using Cuffdiff v2.0.2 (University of Maryland, College Park, MD, USA).

### Luciferase reporter assay

To explore *BFAL1*'s effect on the *RHEB* promoter transcriptional activity, a pGL3-RHEB reporter plasmid was designed and constructed (GENEray Company, Shanghai, China). The pGL3-RHEB plasmid and the relevant *BFAL1* siRNAs or the pCDNA3.1 plasmid were transferred together into cells. To investigate the RHEB 3ʹ UTR activity, three plasmids were designed and synthesized (GENEray): pmirGLO-RHEB 3′ UTR wild-type (WT) plasmid, pmirGLO-RHEB 3ʹ UTR miR-155-5p mut plasmid, and pmirGLO-RHEB 3ʹ UTR miR-200a-3p mut plasmid. Luciferase activity was measured by using a FLUOstar device (Omega Engineering, Deckenpfronn, Germany), with the Dual-Luciferase reporter assay system (Promega).

### Western blotting and antibodies

Proteins were extracted from cells after different treatments and quantified by using a BCA Protein Assay Kit (Thermo Fisher Scientific, West Palm Beach, FL, USA). Proteins (40–60 μg) were electrophoresed through 10% SDS polyacrylamide gels and then electrophoretically transferred onto a polyvinylidene fluoride (PVDF) membrane (Millipore, Bedford, MA, USA). The primary antibodies included those recognizing RHEB (Abcam, Cambridge, UK and Cell Signaling Technology, Danvers, MA, USA), p70S6 Kinase (CST), and Phospho-p70S6 Kinase (CST); β-actin (CST) was used as an endogenous reference. All the secondary antibodies were labeled with horseradish peroxidase (HRP) (Aksomics, Shanghai, China). The signal was detected by using an ECL kit (Pierce Biotech, Rockford, IL, USA).

### ShRNA and adenovirus construction

The shRNAs used in animal experiments and all adenoviruses were constructed and purchased from Obio Technology Company (Shanghai, China): Control shRNA (Y001, Obio), *BFAL1*-shRNA1 (Y2276, Obio), *BFAL1*-shRNA2 (Y9601, Obio), *BFAL1*-overexpressing adenovirus (H8855, Obio), miR-155-5p adenovirus (H9491, Obio), and miR-200a-3p adenovirus (H9492, Obio).

### In vivo experiments

All animal experiments were performed according to the National Institute of Health Guidelines for the Care and Use of Laboratory Animals. Our study was approved by the Animal Care and Use Committee of Renji Hospital, School of Medicine, Shanghai Jiao Tong University. Five-week-old male BALB/c nude mice were obtained from the Experimental Animal Center of Shanghai Institute for Biological Sciences (Shanghai, China). Each mouse was injected with 5.0 × 10^6^ HCT116 cells subcutaneously at the right axilla to establish CRC xenograft tumors. Six days after inoculation, these mice were divided randomly into different groups for different treatments. Different treatments were delivered paratumorally at multiple points every 3 days. The tumor length and width were measured by using calipers every 3 days. The tumor volume was calculated and recorded using the volume formula (long dimension × wide dimension^2^/2). After 21 days, all mice were killed and the subcutaneous xenograft tumors were excised and weighted. Finally, all tumors were kept in formalin for a further marker of proliferation Ki-67 (Ki-67) staining. The tumor volume and weight were presented as mean ± SD (*n* = 5 or 6).

### Statistical analysis

Data were analyzed using Student's *t*-test for comparisons between groups to determine the statistical significance. The Pearson chi-squared test was used to analyze the associations between the patient's clinicopathological characteristics and ETBF abundance or *BFAL1* expression. Kaplan–Meier analysis and the log-rank test were performed to evaluate patient survival. A Cox proportional hazard model was performed to assess the prognostic value of ETBF and *BFAL1*. The difference between the growth rates was determined using analysis of variance (ANOVA) with repeated measures analysis of variances. All statistical tests were performed using SPSS 20.0 statistical software (IBM Corp., Armonk, NY, USA) or Graphpad Prism 5.0 (Graphpad Software Inc., San Diego, CA, USA). Each experiment was repeated at least three times. All data were presented as the mean ± SD and were calculated from three separate experiments. The results were considered statistically significant when the two-tailed *P* value was <0.05.

## Results

### *BFAL1* is highly expressed in ETBF-related CRC tissues and cells

**Fig. 1:**
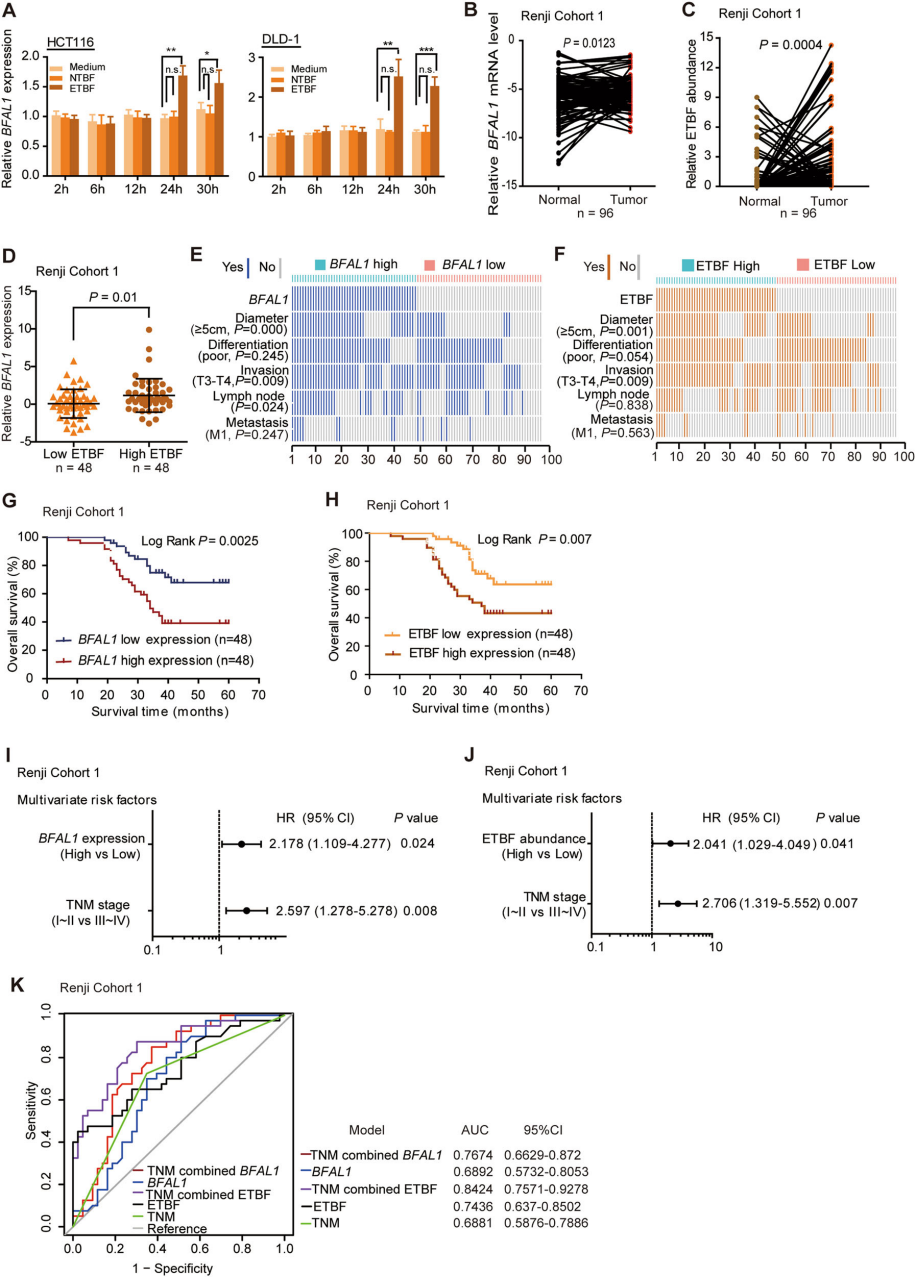
*BFAL1* is upregulated by ETBF and both of them are clinicopathologically related to CRC features and outcomes. **a** The mRNA level of *BFAL1* in ETBF or NTBF-treated HCT116 cells and DLD-1 cells at different time points, compared with cells in a single bacterial medium (mean ± SD of three independent experiments; Student's *t*-test, *P < 0.05, **P < 0.01, ***P < 0.001). **b** Comparison of *BFAL1* mRNA levels in CRC tumor tissues and pair-matched normal tissues in Renji Cohort 1 (*n* = 96, Student's *t*-test, *P* < 0.05). **c** Relative DNA abundance of ETBF in tumor tissues and pair-matched normal tissues, Renji Cohort 1 (*n* = 96, Student's *t*-test, *P* < 0.001). **d** Comparison of *BFAL1* mRNA levels between high ETBF abundance tissues (*n* = 48) and low ETBF abundance tissues (*n* = 48) (Student's *t*-test, *P* < 0.05). **e** Comparing the tumor diameter, pathological differentiation, invasion depth, lymph node involvement, and vascular metastasis between *BFAL1* high and low tumors in Renji Cohort 1. The association of different clinicopathological features was illustrated in a heatmap (statistical significance was performed using the *χ*^2^ test). **f** The correlation of different clinicopathological features with ETBF high- and low-abundance tumors (*χ*^2^ test). **g**Overall survival of patients with CRC patients with high or low *BFAL1* expression in Renji cohort 1, Kaplan–Meier survival analysis (*P* = 0.0025; HR 2.656; 95% CI: 1.409–5.007). **h** Overall survival of patients with CRC with high or low ETBF abundance in Renji Cohort 1, Kaplan–Meier survival analysis (*P* = 0.007; HR 2.351; 95% CI: 1.281–4.462). **i**, **j** Multivariate regression analysis of Renji Cohort 1. **i** included all the CRC clinicopathological factors. **j** Excluded the factor of *BFAL1* expression. **k** ROC analysis based on the ETBF abundance, *BFAL1* expression, and TNM stage in Renji Cohort 1 (bars correspond to 95% confidence intervals)

To identify a specific lncRNA associated with ETBF-related CRC, the top 20 significant CRC-related lncRNAs (tumors vs. normal, false discovery rate (FDR) < 0.01, fold change > 1.5) were targeted in the GEO database GSE20916. After that, these 20 lncRNAs were further filtrated in another GEO database GSE31737 (tumors vs. normal, FDR < 0.01) and finally we got the eight lncRNAs (Fig. S1A). To search for lncRNAs associated with ETBF among these eight candidates, two colorectal cancer cell lines, DLD-1 and HCT116, were treated with ETBF for up to 30 h. Interestingly, significantly increased expressions of lncRNAs AK096729 (*BFAL1*), AK001058, and AK098081 were detected in the ETBF-treated HCT116 and DLD-1 cells after 24 h (Fig. 1a, Fig. S1B), compared with those in NTBF or simple medium-treated cells. This phenomenon indicated that ETBF increases the expression of certain lncRNAs in CRC cells.

To identify the expression profile of these three ETBF-associated lncRNAs in CRC tissues, 96 cases (Renji Cohort 1) were adopted to test both the ETBF abundance and lncRNA expression levels in the cancer tissues paired with noncancerous tissues. The data showed that cancer tissues presented significantly higher expression levels of AK096729 (*P* = 0.0123, Fig. 1b), AK001058 (*P* = 0.0418), and AK098081 (*P* = 0.0289) (Fig. S1C) and simultaneously had a higher abundance of ETBF (*P* = 0.0004, Fig. 1c) compared with those in normal tissues. Furthermore, the correlation between the ETBF abundance and the expression of these lncRNAs in cancer tissues was detected. The results showed that cancer tissues with a relatively high ETBF colonization presented a higher enrichment of AK096729 (*P* = 0.01, Fig. 1d), whereas AK001058 and AK098081 levels were not obviously related with ETBF abundance (Fig. S1D). Therefore, we focused on AK096729 for further study and named it as *Bacteroides fragilis*-associated lncRNA1 (*BFAL1*).

In order to identify whether *BFAL1* is a novel lncRNA, the full-length cDNA of *BFAL1* was isolated by using 3ʹ and 5ʹ rapid amplification of cDNA ends (RACE) and sequenced (Fig. S1E). *BFAL1* is located on chromosome 19 and is mainly distributed in the cell cytoplasm (Fig. S1F). To determine its noncoding character, the *BFAL1* sequence was analyzed by using an ab initio ncRNA transcriptome predictor, which showed that its noncoding probability was 95.22%, whereas its coding probability was 4.78% (Fig. S1G). Analysis of *BFAL1* ORF sequences using a coding potential calculator failed to find any protein-coding potential (Fig. S1H). In addition, we calculated its coding potential using PhyloCSF. The PhyloCSF score of *BFAL1* was –774, indicating that *BFAL1* is unlikely to encode any protein product. Analysis using qPCR showed a higher expression of *BFAL1* in human CRC cells than in normal colorectal epithelial cells (FHC) (Fig. S1I).

### Both ETBF and *BFAL1* are associated with patients' clinicopathology and outcomes

To analyze the relationship between *BFAL1* and the clinicopathological features of CRC, correlation regression analysis was used. Detailed information regarding Renji Cohort 1 is shown in Table S1. The data showed that the expression of *BFAL1* was positively related with tumor size (*P* = 0.000), tumor invasion (*P* = 0.009), and lymph node involvement (*P* = 0.024) (Fig. 1e). Similarly, ETBF abundance also showed a positive relationship with tumor size (*P* = 0.001) and invasion (*P* = 0.009) (Fig. 1f). We further examined the outcomes of these 96 cases. Kaplan–Meier analysis revealed that high expression of *BFAL1* in tumor tissues was associated with a reduced overall survival (*P* = 0.0025; hazard ratio (HR) 2.656; 95% confidence interval (CI) 1.409–5.007) (Fig. 1g). Patients with high ETBF abundance in their tumor tissues also exhibited a poor outcome (*P* = 0.007; HR 2.351; 95% CI 1.281–4.462) (Fig. 1h). Univariate regression analysis showed that both ETBF abundance (*P* = 0.002; HR 2.358; 95% CI 1.230–4.522) and *BFAL1* expression (*P* = 0.004; HR 2.642; 95% CI 1.361–5.129) had the potential to predict CRC prognosis (Fig. S1J). Further multivariate regression analysis showed that *BFAL1* expression (*P* = 0.024; HR 2.178; 95% CI 1.109–4.277) and ETBF abundance (*P* = 0.041; HR 2.041; 95% CI 1.029–4.049) were two independent factors for CRC aggressiveness, with significant hazard ratios for predicting outcomes (Fig. 1i, j). Receiver-operating characteristic (ROC) analysis illustrated that the area under curve (AUC) of the TNM-stage-based model combined with *BFAL1*-based prediction (0.7674) was higher than that of the single TNM-stage model (0.6881), as was the combination of the TNM stage and ETBF prediction (0.8424) (Fig. 1k). This suggested that the combination of *BFAL1* or ETBF and TNM stage was more accurate to predict CRC prognosis than the TNM stage alone.

### *BFAL1* mediates ETBF’s promotion of tumor growth in vitro and in vivo

To evaluate the biological function of *BFAL1* in CRC, we knocked down *BFAL1* using *BFAL1-*siRNA1 in CRC cells and extracted RNA for RNA-seq analysis. A total of 14,737 downregulated genes and 15,913 upregulated genes (≥2-fold) were detected after the knockdown of *BFAL1* in CRC cells (raw data are accessible via the GEO number: GSE129950). Gene set enrichment analysis (GSEA) revealed that the genes related to cell proliferation were reduced (normalized enrichment score (NES) = 1.80, *P* < 0.01; Fig. S2A) and cell cycle-associated pathways were downregulated (NES = 2.80, *P* < 0.01; Fig. S2B) in *BFAL1* knockdown cells. Meanwhile, we also treated DLD-1 cells with ETBF for 24 h and extracted the total human RNA for RNA-seq analysis. We identified 350 upregulated and 154 downregulated genes (≥2-fold and FDR<0.05; raw data accessible via the GEO number: GSE130152). Gene ontology (GO) enrichment analysis also revealed that ETBF treatment regulated a set of genes associated with cell proliferation and the cell cycle (Fig. S2C). These two RNA-seq analyses suggested that both *BFAL1* and ETBF have an effect on CRC cell growth.

**Fig. 2:**
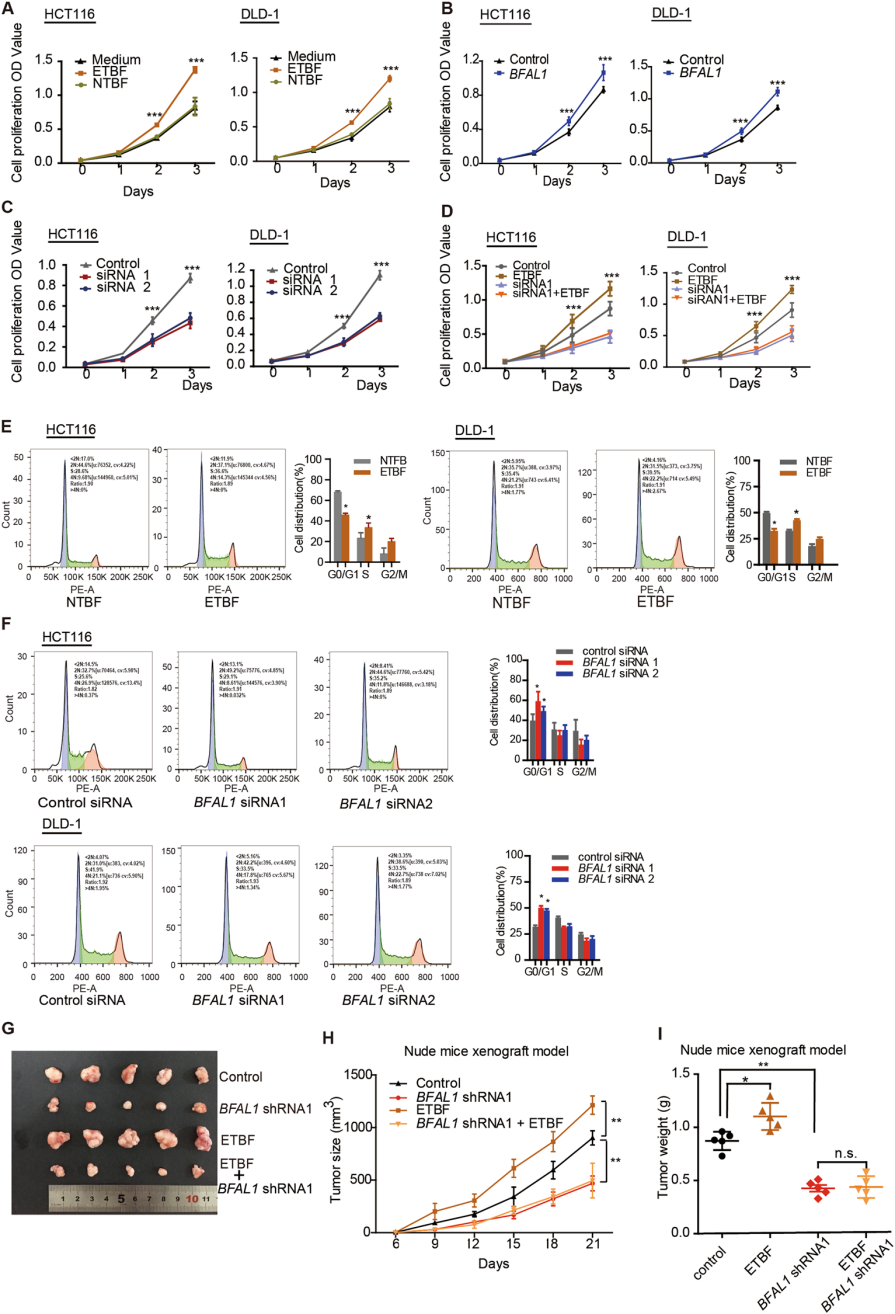
ETBF exerts a biological function on CRC cell growth via *BFAL1* in vitro and in vivo. **a** CCK-8 assay of ETBF-treated HCT116 cells and DLD-1 cells compared with NTBF or single bacterial medium-treated cells (*n* = 6, ANOVA, ***P < 0.001). **b** CCK-8 assay of *BFAL1* overexpression and control cells (*n* = 6, ANOVA, ***P < 0.001). **c** CCK-8 assay of *BFAL1* knockdown in HCT116 cells and DLD-1 cells (*n* = 6, ANOVA, ***P < 0.001). **d** CCK-8 assays of ETBF-treated, *BFAL1* knockdown HCT116 cells and DLD-1 cells (*n* = 6, ANOVA, ***P < 0.001). **e** Cell cycle analysis of ETBF-treated HCT116 cells and DLD-1 cells (mean ± SD of three independent experiments; ANOVA, *P < 0.05). **f** Cell cycle analysis of *BFAL1* knockdown of HCT116 cells and DLD-1 cells (mean ± SD of three independent experiments; ANOVA, *P < 0.05). **g** Xenograft tumors in the nude mouse model under different treatments (*n* = 5). **h** Statistical analysis of tumor sizes (mean ± SD, *n* = 5, ANOVA, **P < 0.01). **i** Tumor weights of different mouse groups (mean ± SD, *n* = 5, ANOVA, *P < 0.05, **P < 0.01)

To validate the biological function of *BFAL1* and ETBF in vitro, CCK-8 assays were performed in HCT116 and DLD-1 cells. The results showed that ETBF enhanced cell proliferation, whereas NTBF and single-medium treatment had no effect on proliferation (Fig. 2a). Meanwhile, we tested the function of *BFAL1* by overexpression from a *BFAL1* plasmid (Fig. S2D) or knockdown with *BFAL1*-siRNA1/2 (Fig. S2E). Overexpression of *BFAL1* promoted cell proliferation (Fig. 2b), whereas *BFAL1* knockdown suppressed cell proliferation (Fig. 2c). Interestingly, ETBF-induced proliferation was significantly suppressed by *BFAL1* knockdown (Fig. 2d), indicating that the ability of ETBF to promote cell proliferation might depend on *BFAL1*. Flow- cytometry assays were performed to test the cell cycle process affected by ETBF and *BFAL1*. The results showed that cell growth was accelerated by ETBF compared with treatment with NTBF (Fig. 2E). In addition, knockdown of *BFAL1* blocked the cell cycle (Fig. 2f).

In vivo, a xenograft tumor model was established in BALB/c nude mice. We observed that ETBF-treated tumors were larger and heavier than those of the control, whereas *BFAL1*-shRNA1/2 virus-treated tumors were smaller and lighter. However, ETBF treatment could not rescue the tumor growth inhibited by *BFAL1*-shRNA1/2 virus treatment (Fig. 2g–i, Fig. S2G–I). Ki-67 staining was consistent with these observations (Fig. S2F, J). From these in vitro and in vivo experiments, we concluded that ETBF exerts its effects on CRC tumor growth through *BFAL1*.

### *BFAL1* mediates ETBF-induced tumor growth by activating the RHEB/mTOR pathway

**Fig. 3:**
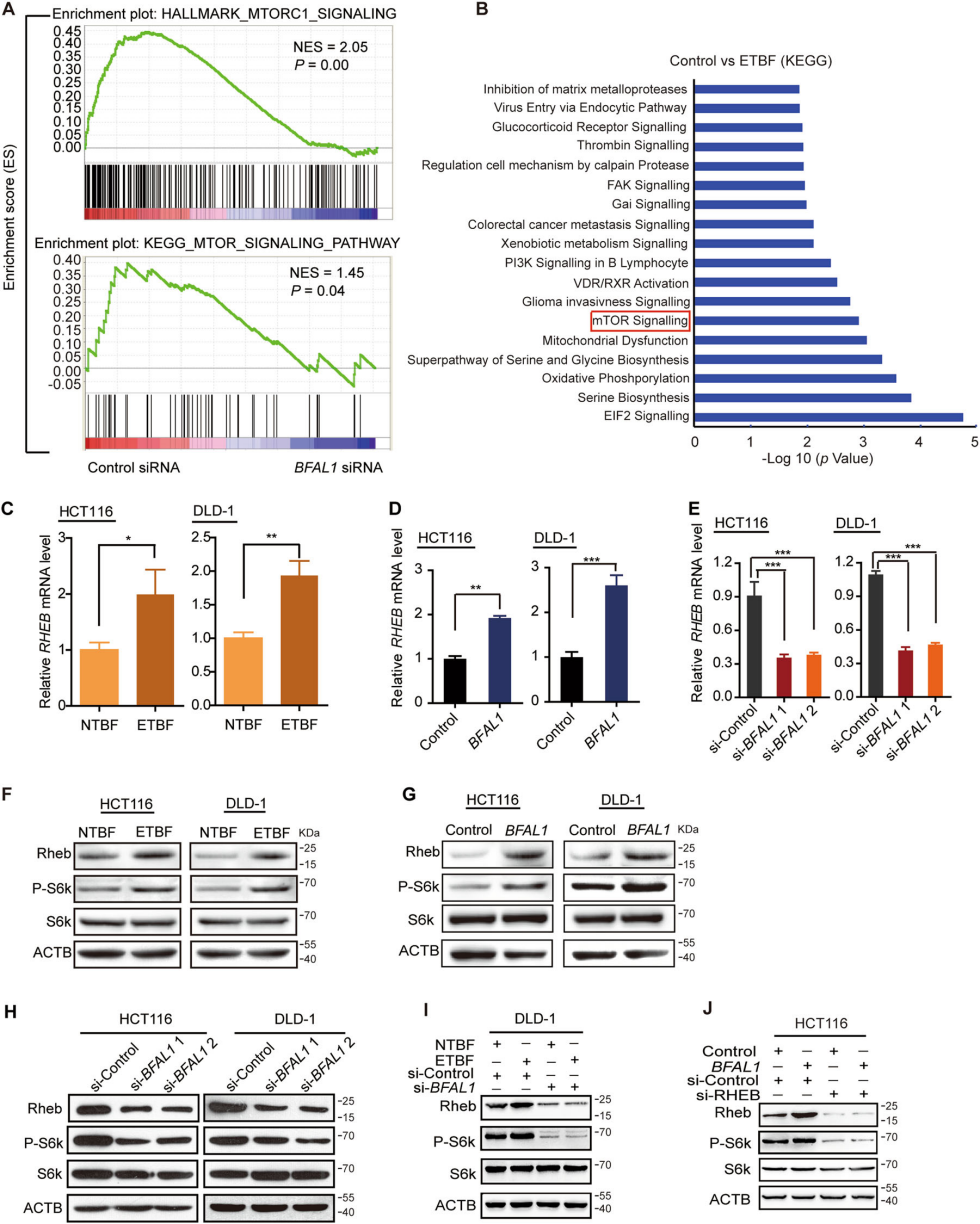
ETBF activates the RHEB/mTOR-signaling pathway via *BFAL1* in CRC. **a** GSEA analysis: enrichment hallmark of mTORC1 signaling (NES = 2.05, *P* = 0.00) and KEGG mTOR-signaling pathway (NES = 1.45, *P* < 0.05). **b** KEGG pathway analysis of ETBF-treated DLD-1 cells. (**c**) The mRNA level of *RHEB* in ETBF-treated HCT116 cells and DLD-1 cells compared with that in NTBF-treated cells. **d** The *RHEB* mRNA level in *BFAL1*-overexpressing HCT116 cells and DLD-1 cells. **e** The *RHEB* mRNA level in *BFAL1-*knockdown HCT116 cells and DLD-1 cells. **f** The protein expression of the RHEB/mTOR pathway in ETBF-treated HCT116 cells and DLD-1 cells, compared with that in NTBF-treated cells. **g** The expression of the RHEB/mTOR pathway in *BFAL1-*overexpressing HCT116 cells and DLD-1 cells. **h** The expression of the RHEB/mTOR pathway in *BFAL1-*knockdown HCT116 cells and DLD-1 cells. **i** The expression of the RHEB/mTOR pathway in DLD-1 cells treated with ETBF after *BFAL1* knockdown. **j** The expression of the RHEB/mTOR pathway in HCT116 cells overexpressing *BFAL1* after *RHEB* knockdown

To further explore the mechanism of *BFAL1* in ETBF-related CRC cell growth, we reviewed the two RNA-Seq data sets mentioned above. Both Hallmark and Kyoto Encyclopedia of Genes and Genomes (KEGG) enrichment analysis in GSEA showed that knockdown of *BFAL1* affected mTOR-associated gene sets (Fig. 3a). KEGG analysis of ETBF-treated cell data also demonstrated that the mTOR-signaling pathway is the significantly functional pathway (Fig. 3b). Taken together, the results suggested that the mTOR signaling is the common downstream pathway of both ETBF and *BFAL1*. We then explored how ETBF and *BFAL1* activate the mTOR pathway. According to the KEGG analysis, the mTOR-signaling pathway in GSEA comprised 12 associated genes affected by *BFAL1* knockdown: *VEGFB*, *EIF4EBP1*, *PGF*, *CAB39L*, *MAPK1*, *FIGF*, *ULK2*, *PIK3CD*, *AKT3*, *EIF4E*, *MLST8*, and *RHEB* (Table S2). To screen out the ETBF and *BFAL1*-targeted gene, we treated DLD-1 cells with ETBF for 24 h and extracted the cell's RNA for gene expression analysis using qPCR. Among the 12 genes, only the *RHEB* mRNA level was upregulated by ETBF (Fig. 3c); the others showed no significant response to ETBF (Fig. S3A). Therefore, *RHEB* became our research focus. RHEB can bind directly to the mTOR complex and regulate the mTOR-signaling pathway by phosphorylating the p70S6 Kinase (S6K);^27^ therefore, we hypothesized that ETBF and *BFAL1* might target *RHEB* to regulate the mTOR pathway. First, we validated the qPCR result from the ETBF-treated cells by showing that overexpression of *BFAL1* upregulated the *RHEB* mRNA level (Fig. 3d), whereas knockdown of *BFAL1* downregulated its level (Fig. 3e). We then confirmed these findings using western blotting. The results demonstrated that the expression levels of RHEB and Phospho-S6k, but not the total S6K, were increased in ETBF-treated cells compared with those in NTBF-treated cells (Fig. 3f). Similar results were obtained in *BFAL1*-overexpressing cells (Fig. 3g). Conversely, the expression levels of RHEB and Phospho-S6k were decreased in *BFAL1*-knockdown cells (Fig. 3h). Furthermore, ETBF could not upregulate RHEB and Phospho-S6K levels after knockdown of *BFAL1* in DLD-1 cells (Fig. 3i). Also, *BFAL1* was not likely to upregulate P-S6K expression after *RHEB* knockdown (Fig. 3j).

### *BFAL1* regulates *RHEB* expression by binding to miR-155-5p and miR-200a-3p

**Fig. 4:**
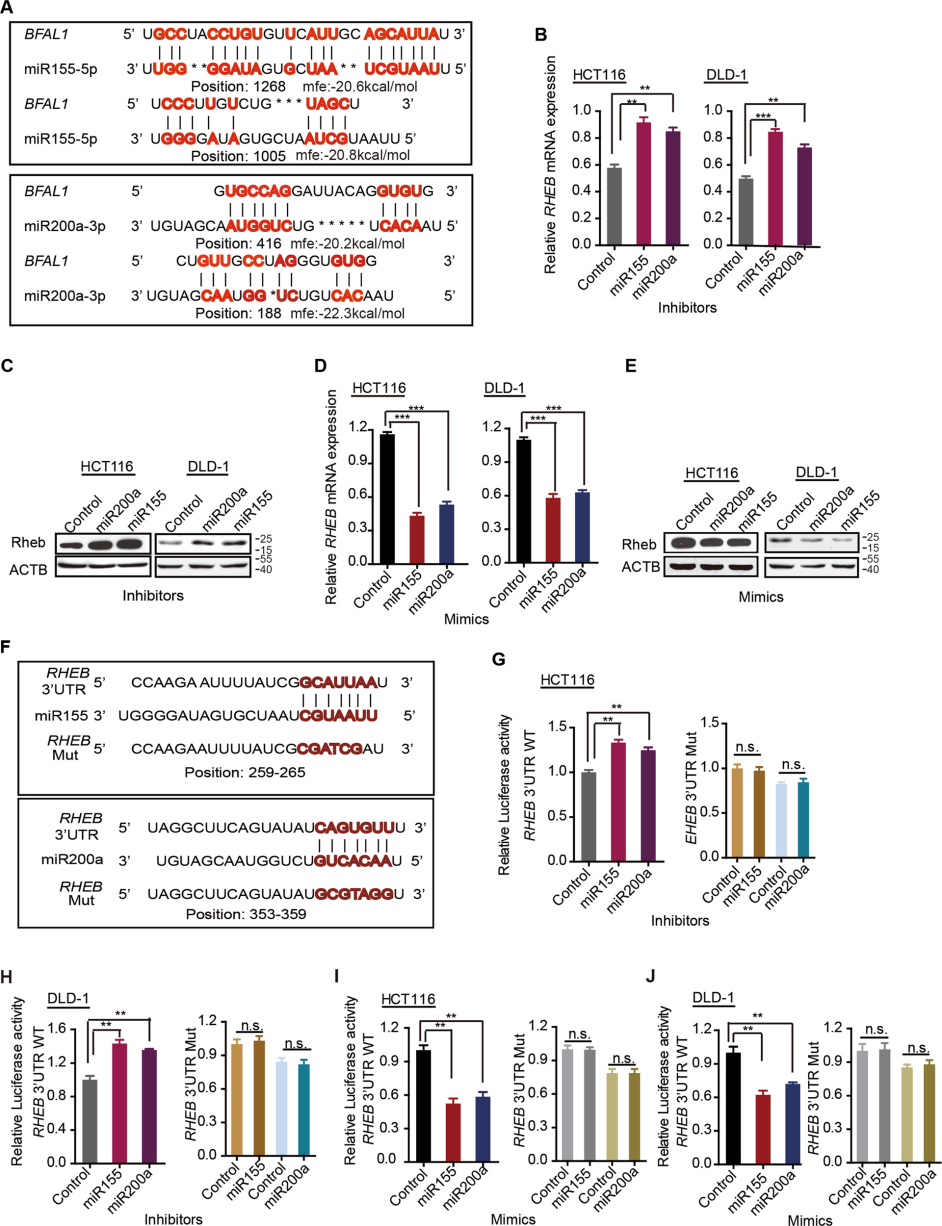
miR-155-5p and miR-200a-3p target the RHEB 3ʹ UTR. **a** The predicted binding sites of miR-155-5p and miR-200a-3p on the *BFAL1* transcripts. **b** *RHEB* mRNA levels in HCT116 cells and DLD-1 cells treated with inhibitors of miR-155-5p or miR-200a-3p. **c** RHEB levels in HCT116 cells and DLD-1 cells transfected with inhibitors of miR-155-5p or miR-200a-3p. **d** *RHEB* mRNA levels in HCT116 cells and DLD-1 cells transfected with mimics of miR-155-5p or miR-200a-3p. **e** RHEB levels in HCT116 cells and DLD-1 cells transfected with mimics of miR-155-5p or miR-200a-3p. **f** The predicted miR-155-5p and miR-200a-3p binding sites on the *RHEB* 3′ UTR and the mutated sites. **g**, **h** Luciferase reporter assays were performed in HCT116 cells and DLD-1 cells transfected with inhibitors of miR-155-5p or miR-200a-3p. The luciferase reporters expressing wild-type or mutant human *RHEB* 3ʹ UTR were used (data represent mean ± SD of three independent experiments, ANOVA, **P < 0.01). **i**, **j** Luciferase reporter assays were performed in HCT116 cells and DLD-1 cells transfected with mimics of miR-155-5p or miR-200a-3p. The luciferase reporters expressing wild-type or mutant human *RHEB* 3ʹ UTR were used (data represent mean ± SD of three independent experiments, ANOVA, **P < 0.01)

We next explored the mechanism of how *BFAL1* regulates the *RHEB* expression. The *RHEB* mRNA level was regulated by *BFAL1* expression; therefore, we synthesized a recombinant luciferase reporter containing the *RHEB* promoter region to test the promoter activity. However, *BFAL1* had no effect on the promoter activity of *RHEB* (Fig. S3B), suggesting that *BFAL1* might regulate *RHEB* mRNA expression via a post-transcriptional mechanism. Recently, it has been reported that miRNAs direct sequence-specific cleavage of the target mRNA and repress its translation, and many RNA transcripts block this activity by sponging miRNA, permitting target mRNA translation as competing endogenous RNAs (ceRNA)^28,29^. The miRNAs that might target *RHEB* were predicted by the TargetScan prediction algorithm^30^ (http://www.targetscan.org) and then were validated in RNAhybrid^31^ (http://bibiserv.techfak.uni-bielefeld.de/rnahybrid) to calculate the complete hybridization around the seed match with the *RHEB* 3′ UTR. Finally, miR-155-5p and miR-200a-3p were screened out, targeting the *RHEB* 3ʹ UTR and also showed high MFE values of hybridization with *BFAL1* (Fig. 4a). Several studies have reported that miR-155-5p and miR-200a-3p target *RHEB* mRNA^32–36^. In the present study, mimics and inhibitory forms of miR-155-5p and miR-200a-3p were synthesized and transfected into CRC cells. Inhibitors of miR-155-5p and miR-200a-3p increased both the mRNA (Fig. 4b) and protein levels (Fig. 4c) of RHEB, whereas their mimics decreased RHEB mRNA and protein levels (Fig. 4d, e). To further test the miRNA effect on the 3ʹ UTR of *RHEB*, pmirGLO-RHEB wild-type plasmids and inhibitors of miR-155-5p and miR-200a-3p were co-transfected into CRC cells. The results showed that the *RHEB* 3ʹ UTR activity was significantly enhanced by the miRNA inhibitors, not the mutated one (Fig. 4f–h). By contrast, mimics of miR-155-5p and miR-200a-3p exerted the opposite effect on *RHEB* 3ʹ UTR activity reporter (Fig. 4i and j). Thus, we confirmed that both miR-155-5p and miR-200a-3p target the 3ʹ UTR of *RHEB* to repress its expression.

**Fig. 5:**
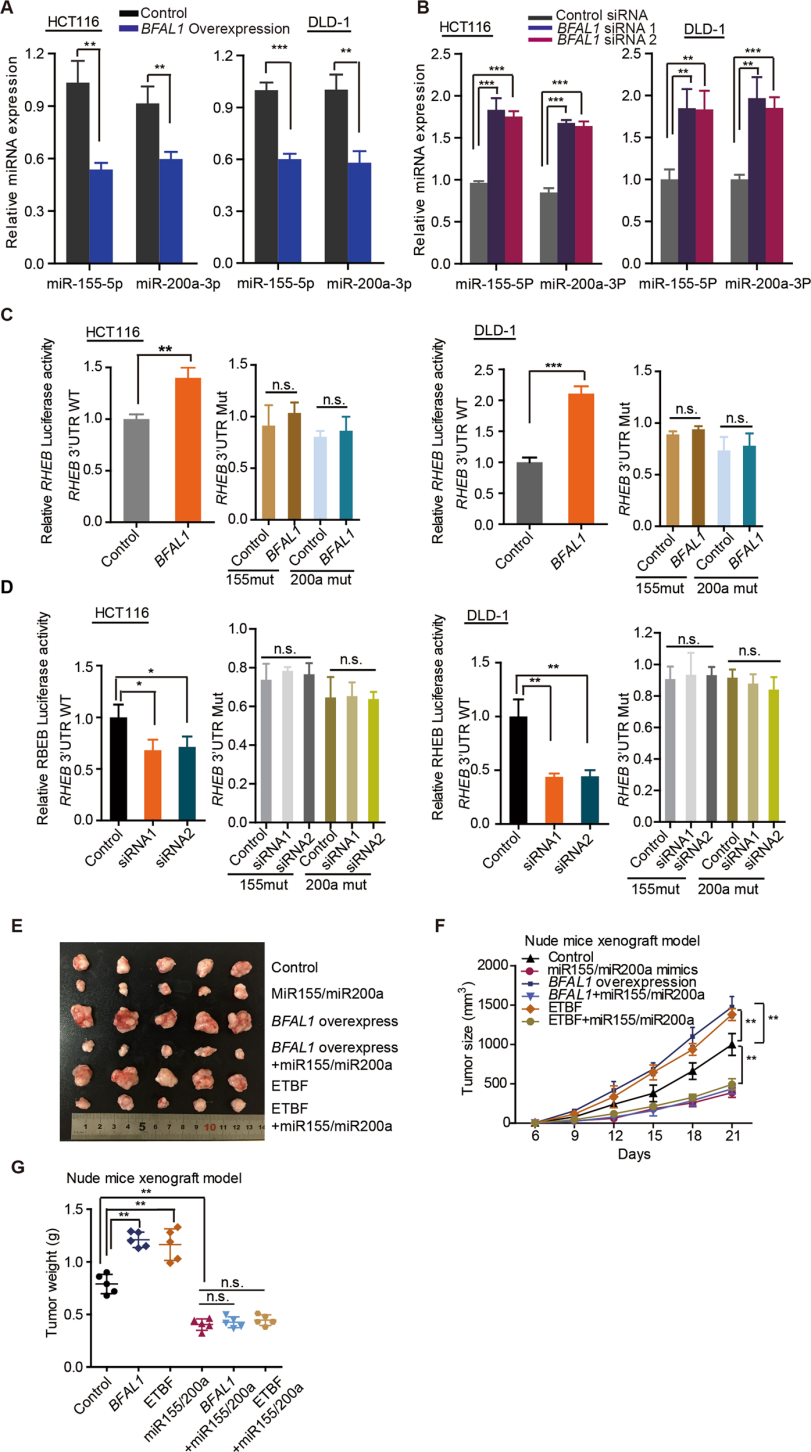
*BFAL1* regulates *RHEB* expression by sponging miR-155-5p and miR-200a-3p. **a** The miR-155-5p and miR-200a-3p mRNA levels in HCT116 cells and DLD-1 cells overexpressing *BFAL1*. **b** The miR-155-5p and miR-200a-3p mRNA levels in HCT116 cells and DLD-1 cells with *BFAL1* knockdown. **c** Luciferase reporter assays were performed in HCT116 cells and DLD-1 cells overexpressing *BFAL1*. Luciferase reporters expressing wild-type or mutant human *RHEB* 3ʹ UTR were used (mean ± SD of three independent experiments, ANOVA, *P < 0.05, **P < 0.01, ***P < 0.001). **d** Luciferase reporter assays were performed in HCT116 cells and DLD-1 cells with *BFAL1* knockdown. Luciferase reporters expressing wild-type or mutant human *RHEB* 3ʹ UTR were used (mean ± SD of three independent experiments, ANOVA, *P < 0.05, **P < 0.01, ***P < 0.001). **e** Xenograft tumors in the nude mouse model under different treatments (*n* = 5). **f** Statistical analysis of tumor sizes (mean ± SD, *n* = 5, ANOVA, **P < 0.01). **g** Analysis of tumor weights in different groups (mean ± SD, *n* = 5, ANOVA, **P < 0.01)

Next, we investigated whether *BFAL1* regulated the *RHEB* expression via miR-155-5p and miR-200a-3p. qPCR analysis showed that overexpression of *BFAL1* decreased miR-155-5p and miR-200a-3p mRNA levels (Fig. 5a), whereas knockdown of *BFAL1* increased their levels (Fig. 5b). Furthermore, luciferase reporter assays revealed that overexpression of *BFAL1* led to activation of the *RHEB* 3′ UTR (Fig. 5c), whereas knockdown of *BFAL1* led to deactivation of the *RHEB* 3ʹ UTR (Fig. 5d). Collectively, the results strongly suggested that *BFAL1* sponged miR-155-5p and miR-200a-3p to regulate *RHEB* expression acting as a ceRNA^37,38^.

In vivo, we also confirmed that miR-155-5p and miR-200a-3p had biological functions using a xenograft tumor model. The results showed that transfection with viruses expressing miR-155-5p and miR-200a-3p obviously attenuated tumor growth compared with the controls. Although *BFAL1*-overexpressing virus and ETBF treatment enhanced tumor growth, they could not entirely recover the growth retardation induced by the miR-155-5p and miR-200a-3p mimics (Fig. 5e–g, Fig. S3C). Collectively, these results confirmed that *BFAL1* binds competitively with miR-155-5p and miR-200a-3p to attenuate their suppressive function on *RHEB* expression.

**Fig. 6:**
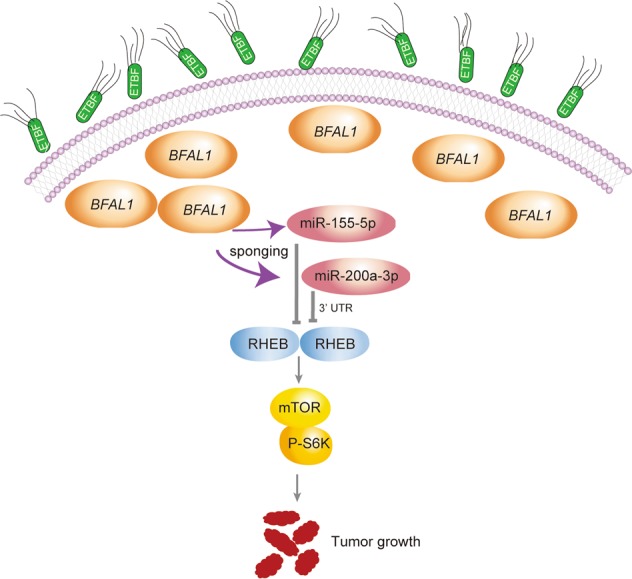
Schematic diagram of ETBF–BFAL1 functions in CRC tumor growth. ETBF may stimulate *BFAL1* overexpression, which competitively binds with miR-155-5p and miR-200a-3p, resulting in the activation of the *RHEB*/mTOR pathway, ultimately promoting CRC tumor growth

In conclusion, we highlighted a new signaling cascade of ETBF–*BFAL1*–RHEB/mTOR that promotes tumor growth in CRC (Fig. 6).

## Discussion

The gut microbiota, as a complicated symbiotic organ in mammals, has marked effects on host health and disease. The gut microbiome provides environmental cues to which the host responds via alterations in the host epigenome and gene expression^22^. Previous studies have focused on the roles of protein-coding genes. However, increasing evidence has demonstrated a crosstalk between the microbiota and epigenetic alterations^39^. DNA methylation, histone modifications, and various noncoding RNAs are involved in bacteria-related cancers^40,41^. However, the regulatory mechanism remains largely unknown. ETBF is regarded as one of the most prominent bacterial species in human CRC. Although it has been studied for many years, the mechanism by which ETBF alters the host's noncoding RNAs remains poorly understood. Through a series of genomic, bioinformatic, biological, xenograt model, and clinical studies, we identified that lncRNA *BFAL1* is upregulated by ETBF and revealed a new mechanism by which *BFAL1* participates in ETBF-induced CRC formation.

A study from Turkey has examined ETBF in a CRC population. Using *bft* as a marker, ETBF was detected more frequently in the stools of consecutive cases of CRC compared with that in concurrent hospital-based, age-, and gender-matched patients without CRC (38% ETBF in 73 cases of CRC and 12% ETBF in 59 controls, *P* < 0.01)^42^. In the present study, we demonstrated a higher abundance of ETBF in CRC tissues compared with that in pair-matched normal tissues. Furthermore, we revealed high expression of *BFAL1* in cancer tissues, which correlated positively with ETBF abundance. These findings indicated that direct exposure of colorectal epithelial cells (CECs) to ETBF may result in the upregulation of certain lncRNAs, including *BFAL1* in CECs. In CRC cells, we observed that the *BFAL1* mRNA level was upregulated by ETBF treatment. Therefore, we hypothesized that *BFAL1* is responsible for ETBF stimulation of CRC and focused on *BFAL1* for further study.

In a clinical study, we found that ETBF and *BFAL1* have similar clinicopathological effects on tumor size. In addition, patients with high levels of ETBF and *BFAL1* expression had a poor prognosis. These findings strongly suggested the emerging prognostic and therapeutic value of ETBF and *BFAL1*.

Initiation and progression of CRC refers to events yielding biological changes that foster CEC proliferation and multiple gene mutations or epigenetic alterations, ultimately resulting in the transformation to cancer. However, the events that precipitate in this process remain unknown. The microbiome is a prime suspect for triggering the initiation and/or progression of CRC carcinogenesis^7^. Recent studies have demonstrated that the gut microbiota exerts various biological functions on tumor formation and progression. *Fusobacterium nucleatum* was confirmed to induce autophagy and mediate CRC chemoresistance^27^, and is also associated with metastasis of primary CRC^43^. The present study revealed that ETBF exerts a marked effect on CRC tumor growth in vitro and in vivo. However, when *BFAL1* was knocked down, ETBF lost its biological effect on CRC, suggesting that *BFAL1* mediates ETBF’s carcinogenic function in CRC.

Further mechanistic investigations revealed a hitherto unidentified signaling cascade of ETBF–*BFAL1* RHEB/mTOR in CRC carcinogenesis. The mTOR-signaling pathway is reported to be dysregulated in ~50% of all human malignancies. This pathway acts as a sensor that integrates intracellular and extracellular events concerning metabolism and nutrients, and coordinates cell growth and autophagy^44^. RHEB is a regulator of mTOR and is indispensable for mTOR activation in response to all stimuli^45^. RHEB binds to mTOR on the lysosome surface and inactivates downstream signaling^46^. mTOR signaling is rarely reported in microbiota-associated CRC issues. Our findings revealed the exact mechanism of the ETBF-induced mTOR pathway mediated by *BFAL1*. *BFAL1* regulates *RHEB* expression by binding competitively to miR-155-5p and miR-200a-3p, potentially acting as a ceRNA. Recent studies have provided evidence that gut microbiota might modulate miRNAs to exert biological functions. For example, *F. nucleatum* might modulate the expression of miR-18* and miR-4082^27^, and *Escherichia coli* might modulate miR-30C and miR-130A to regulate human colonic epithelial cell autophagy^47^. By contrast, lncRNAs have been reported to bind with particular miRNAs to attenuate their suppressive effect on the target gene^48,49^. For example, precise regulation by lncRNA *uc.173* was demonstrated in the control of gut permeability by decreasing the availability of miR-29b to regulate *CLDN1* mRNA expression^50^. In the present study, we confirmed that *BFAL1* regulates *RHEB* expression by binding to miR-155-5p and miR-200a-3p in ETBF-associated carcinogenesis.

Taken together, ETBF orchestrates the *BFAL1*, miR-155-5p/miR-200a-3p, and the RHEB/mTOR pathways to regulate CRC tumor growth. LncRNA *BFAL1* might be a promising target for CRC diagnosis and therapy.

## Supplementary information

Supplementary Figure S1

Supplementary Figure S2

Supplementary Figure S3

Supplementary Table S1

Supplementary Table S2

Supplementary Material Table

WB original figures

Supplementary figure legends

## References

[1] Labianca R (2010). Colon Cancer. Crit. Rev. Oncol./Hematol..

[2] Smith RA, Cokkinides V, Brooks D, Saslow D, Brawley OW (2010). Cancer screening in the United States, 2010: a review of current American cancer society guidelines and issues in cancer screening. CA: A Cancer J. Cilni..

[3] Siegel R, Desantis C, Jemal A (2014). Colorectal cancer statistics, 2014. CA: A Cancer J. Clini..

[4] Terzic J, Grivennikov S, Karin E, Karin M (2010). Inflammation and colon cancer. Gastroenterology.

[5] Viaud S (2013). The intestinal microbiota modulates the anticancer immune effects of cyclophosphamide. Science.

[6] Vetizou. (2015). Anticancer immunotherapy by CTLA-4 blockade relies on the gut microbiota. Science.

[7] Sears CL, Garrett WS (2014). Microbes, microbiota, and colon cancer. Cell Host Microbe.

[8] DeStefano Shields CE (2016). Reduction of murine colon tumorigenesis driven by Enterotoxigenic Bacteroides fragilis using cefoxitin treatment. J. Infect. Dis..

[9] Peloquin JM, Nguyen DD (2013). The microbiota and inflammatory bowel disease: insights from animal models. Anaerobe.

[10] Liam Chung. (2018). Bacteroides fragilis toxin coordinates a procarcinogenic inflammatory cascade via targeting of colonic epithelial cells. Cell Host Microbe.

[11] Wu S, Dreyfus LA, Tzianabos AO, Hayashi C, Sears CL (2002). Diversity of the metalloprotease toxin produced by Enterotoxigenic Bacteroides fragilis. Infect. Immun..

[12] Sears CL, Geis AL, Housseau F (2014). Bacteroides fragilis subverts mucosal biology from symbiont to colon carcinogenesis. J. Clin. Investig..

[13] Hyum CR, Do YY, Su HK, Young JK, Jung MK (2011). *Bacteroides fragilis* enterotoxin upregulates intracellular adhesion molecule-1 in endothelial cells via an eldose reductase-, MAPK-, and NF-κB-Dependent pathway, leading to monocyte adhesion to endothelial cells. J. Immunol..

[14] Kun Z (2011). The lver-enriched lnc-LFAR1 promotes liver fibrosis by activating TGFβ and Notch pathways. Nat. Commun..

[15] Francesco PM, Ivan R, Maite H (2017). The multidimensional mechanisms of long noncoding RNA function. Genome Biol..

[16] Prensner JR, Chinnaiyan AM (2011). The emergence of lncRNAs in cancer biology. Cancer Discov..

[17] Yang F (2011). Long noncoding RNA high expression in hepatocellular carcinoma facilitates tumor growth through enhancer of zeste homolog 2 in humans. Hepatology.

[18] Kretz M (2013). Control of somatic tissue differentiation by the long non-coding RNA TINCR. Nature.

[19] Yoon JH, Abdelmonhsen K, Gorospe M (2013). Posttranscriptional gene regulation by long noncoding RNA. J. Mol. Biol..

[20] Quinn JJ, Chang HY (2016). Unique features of long non-coding RNA biogenesis and function. Nat. Rev. Genet..

[21] Yufeng Q, Paul AW (2018). Crosstalk between the microbiome and epigenome messages from bugs. J. Biochem..

[22] Nicholson JK (2012). Host-gut microbiota metabolic interactions. Science.

[23] Paul B (2015). Influences of diet and the gut microbiome on epigenetic modulation in cancer and other diseases. Clin. Epigenetics.

[24] Liang LX, Ai LY, Qian J, Fang JY, Xu J (2015). Long noncoding RNA expression profiles in gut tissues constitute molecular signatures that reflect the types of microbes. Sci. Rep..

[25] Pantosti A, Melpeli M, Wilks M, Menozzi MJ, D'Ambrosio (1997). Detection of enterotoxigenic Bacteroides fragilis by PCR. J. Clin. Microbiol..

[26] Antunes ENF (2002). Pattern III non-toxigenic Bacteroides fragilis (NTBF) strains in Brazil. Anaerobe.

[27] Xiaomeng L, Yenshou L, Sara OV, Kazuyoshi Y, Joseph A (2005). Rheb binds and regulates the mTOR kinase. Curr. Biol..

[28] Cesana M (2011). A long noncoding RNA controls muscle differentiation by functioning as a competing endogenous RNA. Cell.

[29] TaChung Y (2017). Fusobacterium nucleatum promotes chemoresistance to colorectal cancer by modulating autophage. Cell.

[30] Lewis BP, Burge CB, Bartek DP (2015). Conserved seed pairing, often flanked by adenosines, indicates that thousands of human genes are microRNA targets. Cell.

[31] Jan K, Marc R (2016). RNAhybrid: microRNA target prediction easy, fast and flexible. Nucleic Acids Res..

[32] Lv J (2017). Ox-LDL-induced microRNA-155 promotes autophagy in human endothelial cells via repressing the Rheb/mTOR pathway. Cell Physiol. Biochem..

[33] Xing G (2016). Influence of miR-155 on cell apoptosis in rats with ischemic stroke role of the ras homolog enriched in brain (Rheb)/mTOR pathway. Med Sci. Monit..

[34] Caballero GE (2015). In vivo inhibition of miR-155 promotes recovery after experimental mouse stroke. J. Neurosci..

[35] Wang Y (2017). MicroRNA-199a-5p induced autophage and inhibites the pathogenesis of ankylosing spondylitis by modulating the mTOR signaling via directly targeting Ras Homolog Enriched in Brain (Rheb). Cell Physiol. Biochem.

[36] Guo W (2016). miR-200a regulates Rheb-mediated amelioration of insulin resistance after duodenal-jejunal bypass. Int J. Obes. (Lond.).

[37] Qian K (2018). Identifying autophage gene-associated module biomarkers through constrcution and analysis of an autophage- mediated ceRNA-ceRHA interaction network in colorectal cancer. Int J. Oncol..

[38] Zhu H, Zheng T, Yu J, Zhou L, Wang L (2018). LncRNA XIST accelerates cervical cancer progression via upregulating fus through competitively binding with miR-200a. Biomed. Pharmacother..

[39] Bultman SJ, Jobin C (2014). Microbial-derived butyrate: an oncometabolite or tumor-suppressive metabolite?. Cell Host Microbe.

[40] Wu X, Zhang Y (2017). TET-mediated active DNA demethylation: mechanism, function and beyond. Nat. Rev. Cenet..

[41] Feil R, Frage MF (2012). Epigenetics and the environment: emerging patterns and implications. Nat. Rev. Genet..

[42] Toprak NU (2006). A possible role of Bacteroides fragilis enterotoxin in the aetiology of colorectal cancer. Clin. Microbiol. Infect..

[43] Susan B (2017). Analysis of *Fusobacterium* persistence and antibiotic response in colorectal cancer. Science.

[44] Zhang YJ, Duan Y, Zheng XF (2011). Targeting the mTOR kinase domain: the second generation of mTOR inhibitors. Drug Discov. Today.

[45] Wang XW, Zhang YJ (2014). Targeting mTOR network in colorectal cancer therapy. World J. Gastroenterol..

[46] Sang GK, Gwen RB, John B (2013). Nutrient regulation of the mTOR Complex 1 signaling pathway. Mol. Cells.

[47] Nguyen HT, Dalmasso G, Muller S, Darfeuille M (2014). Crohn's disease-associated adherent invasive *Escherichia coli* modulate levels of microRNAs in intestinal epithelial cells to reduce autophage. Gastroenterology.

[48] Quan H (2018). LncRNA-AK13185 sponges MiR-93-5p in newborn and mature osteoclasts to enhance the secretion of vascular endothelial growth factor a promoting vasculogenesis of endothelial progenitor cells. Cell. Physiol. Biochem..

[49] Yuan JH (2014). A long noncoding RNA activated by TGF-β promotes the invasion-metastasis cascade in hepatocellular carcinoma. Cancer Cell.

[50] Wang JY (2018). Regulation of intestinal barrier function by lncRNA *uc.173* through interaction with miR-29b. Mol. Cell. Biol..

