# Supplementary material for: Long noncoding RNA BFAL1 mediates enterotoxigenic Bacteroides fragilis-related carcinogenesis in colorectal cancer via the RHEB/mTOR pathway
Source: Cell Death Dis. 2019 Sep 12;10(9):675. doi: 10.1038/s41419-019-1925-2 (PMC6742644; doi:10.1038/s41419-019-1925-2)

Supplementary Figure S1

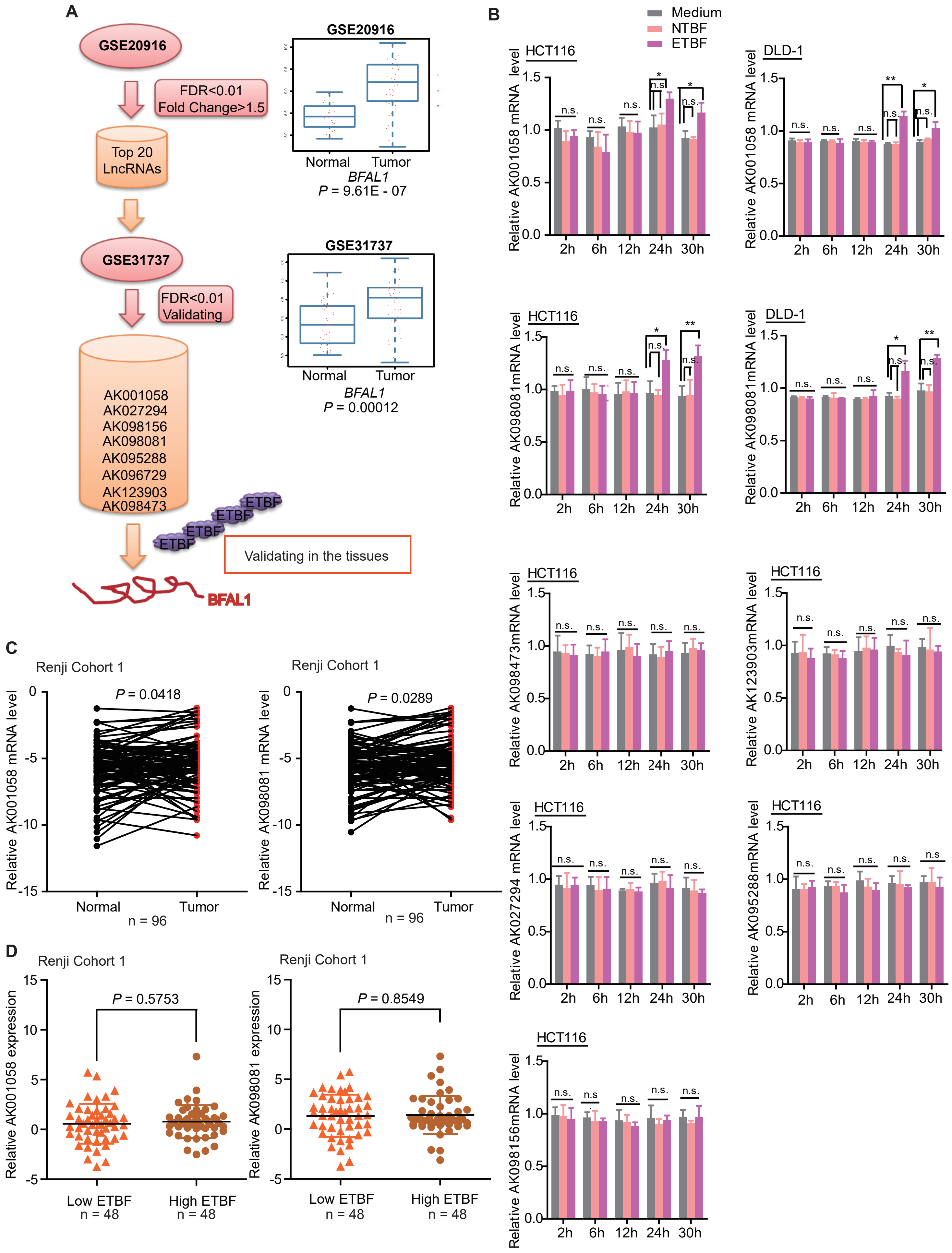

Supplementary Figure S1

E

***BFAL1* sequence**

TCCTGGGCTCAAGCGATCCTCCCACCTGAGTCTCCTGAGTACCTGGGACC
ACAGGCACATGCTAACGCACCCACTTAATTTTTTTATTCGTAGAGCCGAG
GTCTCACTATGTTGCCCAGACTGGTCTCAAGCTCTTGGGCTCAAGCAGTC
CTCCTGCCTCAGCCTCCCAAAGTGCCAGGATTACAGGTGTGAGCCACCAC
ACCCAGTCCAAAAAGTGATTTTCTAATTCCATAGTTTCTTCTACATTTCT
TAACTGGAATTTTGTAAGGAAGAGTTATCCTTATTCATTTATTTACCCAT
TTACTTGTAGGAGCATGTATCATAAATTCTTAATCTATGGGGTGTAGTTA
GCTACTGTAAATTTGATGCACATATTTTTCCAGATTCAATAAGTAGGATC
CCCATCAAGCTATCTTCTGAATCTTTAAAAATGTATTCTCCTTATTTCAT
GGGTATATAATTATTTTCTGGGCAACAGTTTGTTCATGGTACATTTGTCT
CCAGCCCTAGACTCAGAAAAATGTAATTTATTTTAATGGAGAATACTATT
TAGAAGCTGCAGTCTCTGTGTAGGGAGGTGTTCCTAGGTAATAGTGCCCT
GGCTTTTAGGTCTTCTCTATGGAGGATATGGCAAAGACATACAAAATTAT
ATATACGTATCCACACATTAATAACTCTATTTCTATATTAAAAACATGAC
CACAAGCCAACACTTTTAATTCCACTGCAGCACTGCTGGGTTATTTCTTG
CTTTCCAAATTCCCTTGTCTGTAGCTATCTTTCATCACTTGCAAAAATAG
CACGTGCTCAATCCTAGATTAAAATATAAGCTACTCTCAGAATTGATAAC
TCATACCACTCTTTTTAAAAATCAACTAACCAGAATTCAGTTTTTATTCA
CAGTTCTTATCTTTAGCTCAGGGCGTAATATAAAAGCACTAGGTTTAAAA
CTTACTTGGTCAGCAGCAATCCACTTCTAGGTACATGTCCAAAGGAACTG
AAGAAAGCATCTCAATGTGTGAATGCCTACCTGTGTTCATTGCAGCATTA
TTCAGAAGAGCCAAGAGGTGGAAGCAAAGTAAATGTCCATTGACAGATGA
ATTGGTAAACAAAATGCATTATATACATACAATGCAATATCATTTAGCCT
TAAAAACAAAATTCTGCCATATGCTACAACATGGACGTTATGCTAAGTGA
AATAAACCAG

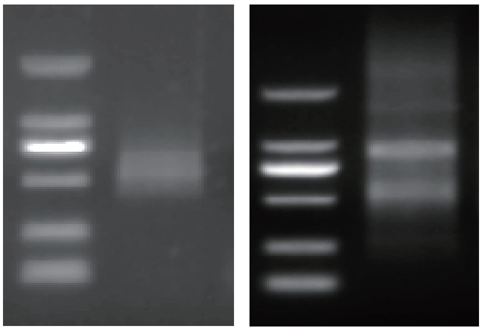

3'RACE 5'RACE

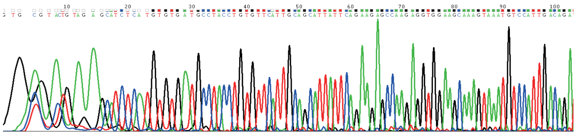

3'sequence

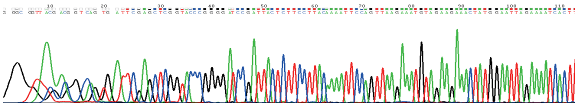

5'sequence

F

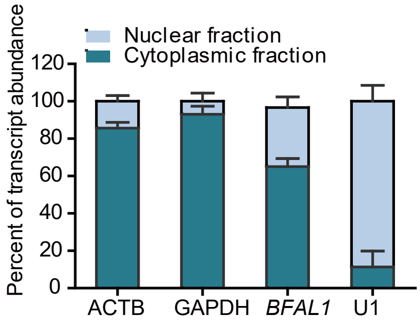

G

Ab Inito ncRNA transcriptome predictor

Checking and correcting sequence...
Number of sequences to be processed: 1
Predicting ncRNA.../work/ncRNA/web/ncRNA_180611744340829/Input_180611744340829.tx

Sequence name	Coding Probably	Non-coding Probably
AK096729.1	4.78%	95.22%

H

HOMOLOGY FEATURES	HIT NUM	3	
	HIT SCORE	11.365636671002	
	FRAME SCORE	43.059232312413	
ORF_FRAMEFINDER	COVERAGE	5.74 %	
	LOG-ODDS SCORE	29.77	
	TYPE	Full	

legend: non-coding ——— coding

I

Human colorectal cancer cell lines

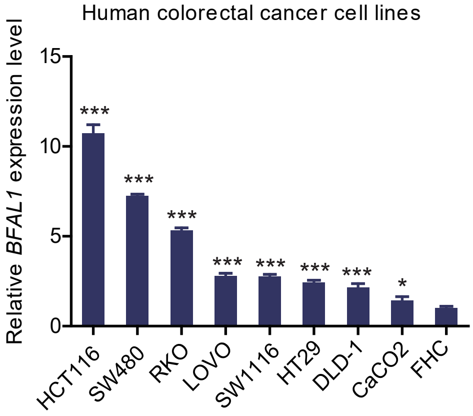

J Renji Cohort 1

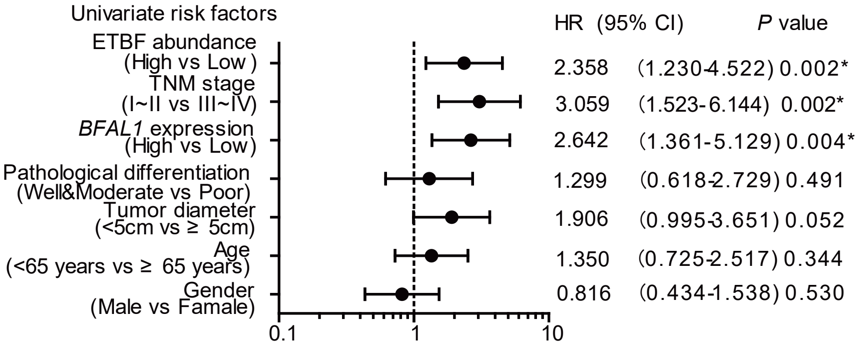

Supplement: Supplementary file 1 — Supplementary Figure S1 [file 41419_2019_1925_MOESM1_ESM.pdf]
