# Supplementary figures and images for: Long noncoding RNA BFAL1 mediates enterotoxigenic Bacteroides fragilis-related carcinogenesis in colorectal cancer via the RHEB/mTOR pathway
Source: Cell Death Dis. 2019 Sep 12;10(9):675. doi: 10.1038/s41419-019-1925-2 (PMC6742644; doi:10.1038/s41419-019-1925-2)

Supplementary Figure S2

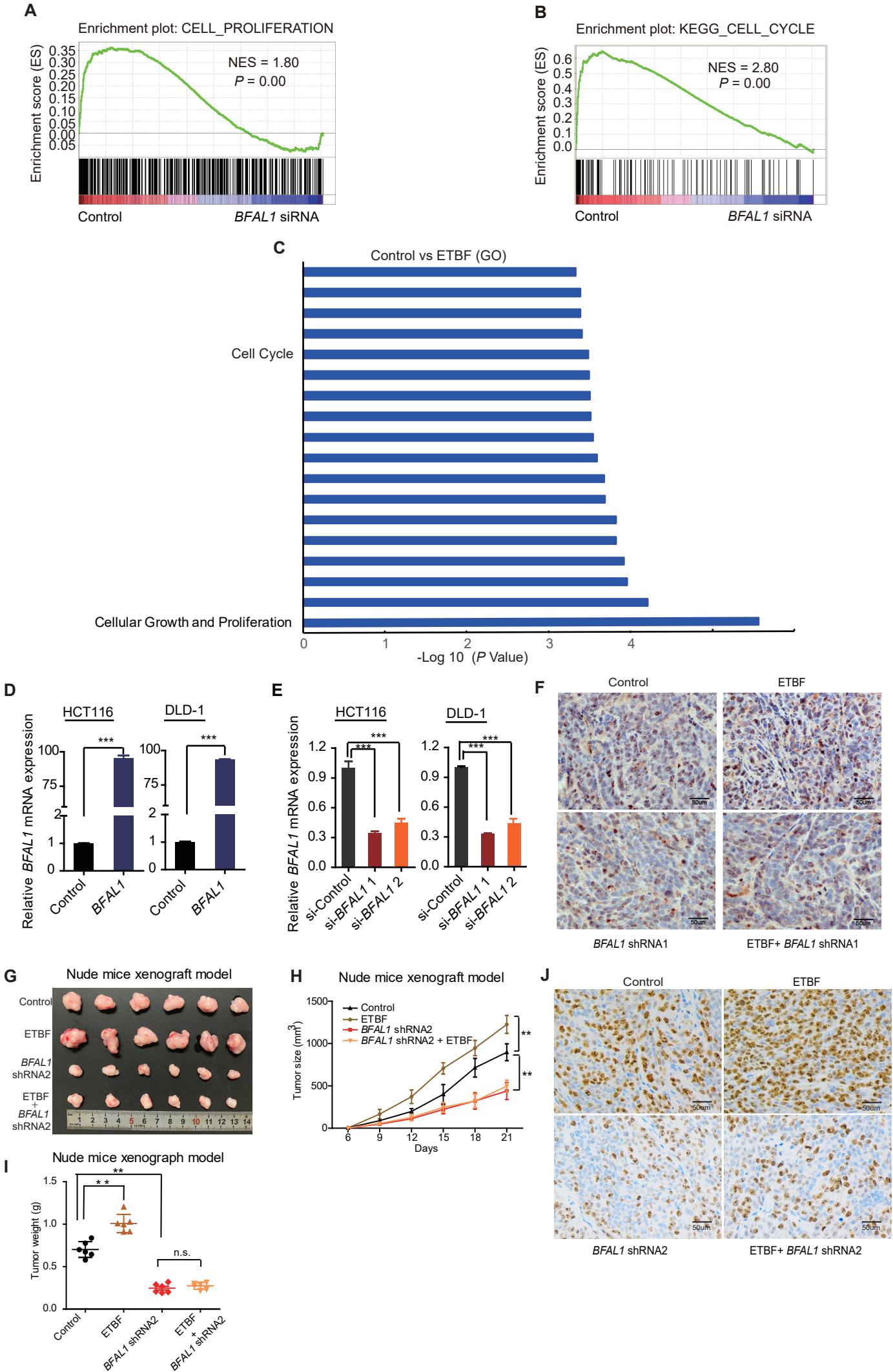

Supplement: Supplementary file 2 — Supplementary Figure S2 [file 41419_2019_1925_MOESM2_ESM.pdf]

Supplementary Figure S3

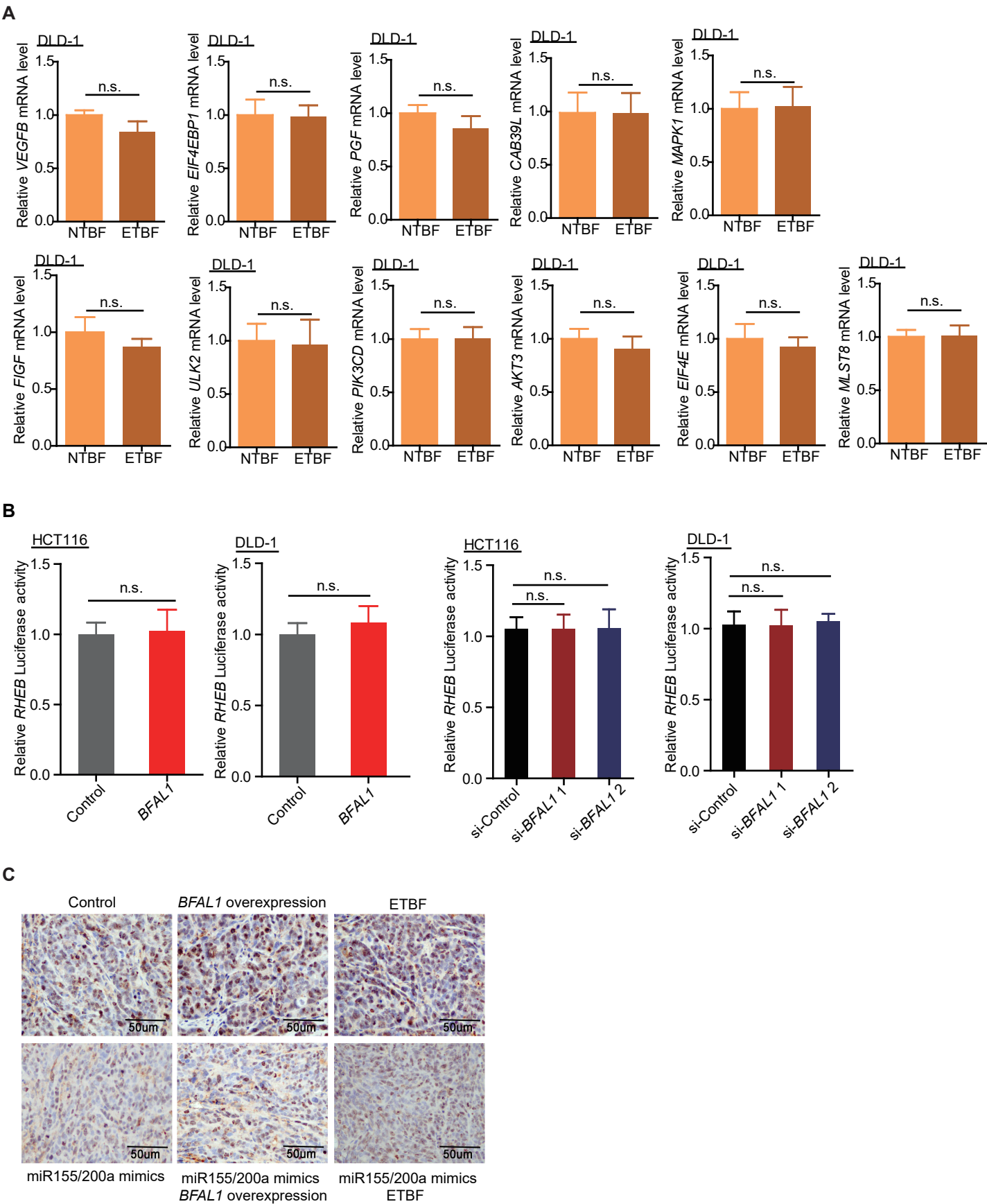

Supplement: Supplementary file 3 — Supplementary Figure S3 [file 41419_2019_1925_MOESM3_ESM.pdf]
