# Supplementary material for: Long noncoding RNA BFAL1 mediates enterotoxigenic Bacteroides fragilis-related carcinogenesis in colorectal cancer via the RHEB/mTOR pathway
Source: Cell Death Dis. 2019 Sep 12;10(9):675. doi: 10.1038/s41419-019-1925-2 (PMC6742644; doi:10.1038/s41419-019-1925-2)
Supplement: Supplementary file 4 — Supplementary Table S1 [file 41419_2019_1925_MOESM4_ESM.pdf]

Supplementary Table S1: Clinical information of 96 cases CRC patients

Gender	Age	Tumor size (cm)	TNM Stage	Survival	Status
Male	66	3.5*3*0.5	T3N0M0	60	Alive
Male	77	5*3*0.5	T4N1M0	60	Alive
Male	30	5.5*2.5*1	T4N2M0	59	Alive
Male	85	5.5*5*0.5	T4N1MO	60	Alive
Female	78	2*2*0.5	T3N0M1	39	Dead
Male	48	5*2*1	T4N2M0	19	Dead
Male	55	5*4*0.5	T4N0M0	59	Alive
Male	71	1.5*1*0.5	T4N0M0	59	Alive
Male	61	5.5*3*1	T3N1M1	29	Dead
Female	39	7*5*5	T4bN0M0	59	Alive
Female	64	4*3*2	T3N0M0	58	Alive
Male	68	7.5*5*1.5	T4N0M0	38	Dead
Female	72	2*2	T1N0M0	58	Alive
Male	49	5*3*1.5	T4N0M1	33	Dead
Male	75	4*3*1	T3N0M0	57	Alive
Male	40	5*4*1	T3N0M0	57	Alive
Male	50	5.5*2.5*1.5	T3N0M0	57	Alive
Male	69	4*4*1	T2N0M0	55	Alive
Male	48	4.5*2*1	T4N2M0	35	Dead
Male	79	5*4*1	T4N0M0	19	Dead
Female	51	6*4.5*1	T3N1M0	57	Alive
Male	74	5*4*1	T4N0M0	21	Dead
Male	44	5*4*1	T4N0M0	39	Alive
Male	74	5.5*4*1	T4N1M0	39	Alive
Male	55	5.5*3.5*0.5	T3N0M0	43	Alive
Male	51	5.5*4*1	T4N1M1	33	Dead
Male	61	9*6*1	T4N2M0	41	Alive
Female	82	8*2*1.5	T3N0M0	41	Alive
Male	64	3*2*0.5	T4N1M0	34	Dead
Female	61	6*5*1	T3N0M1	38	Dead
Female	81	9*3.5*3	T4N2M1	21	Dead
Male	65	2*2*1	T3N1M0	23	Alive
Male	62	4*3*1	T3N1MO	38	Alive
Female	65	3*3*1.8	T4anN1M0	33	Dead
Female	67	4*4*0.8	T4N1M1	41	Dead
Male	46	6*4*1.2	T4N1M0	22	Dead
Female	77	4.5*4.5*1	T2N1M0	24	Dead
Male	78	4.5*4*1	T2N0M0	45	Alive
Male	73	5*4*0.5	T4N0M0	23	Dead
Male	55	5*2*1	T4aN0M0	37	Alive
Female	49	2*2*0.5	T2N2aMO	31	Dead
Female	85	4*3*1	T3N2M0	27	Dead
Male	55	1*1*1	T2N0M0	60	Alive
Female	79	4*3*1	T3N0M0	37	Alive
Female	84	4*4*2	T4aN2aM0	37	Alive
Male	67	5*3.5*0.7	T4aN2M1	23	Dead
Female	59	4*3.5*0.7	T4N0M0	34	Dead
Male	61	8*6*2	T4N2M0	44	Alive
Female	80	5*4*1.5	T3N0M0	43	Alive
Female	52	2*2*1.3	T2N0M0	42	Alive
Male	88	5*3.5*1	T4aN0M0	26	Dead
Male	55	3*3*1	T4N1M0	42	Alive
Female	86	5.5*3*1.5	T4N1M1	25	Dead
Male	62	5*3*1	T4aN1M0	42	Alive
Male	52	2.5*2.5*1	T2N0M0	42	Alive
Female	64	2.8*2.5*1	T2N0M0	40	Alive
Female	60	5*2*1.5	T4aN0M0	23	Dead
Male	77	5*4*1	T2N0M0	41	Alive
Female	25	6*3*1.5	T4N2M0	34	Dead
Male	65	5	T4N0M0	21	Dead
Male	58	3.5*3.5*2	T2N0M0	41	Alive
Male	62	6*5*1	T4N2M1	11	Dead
Male	67	4*3*1	T2N0M0	41	Alive
Male	72	6*5*1	T4N1M0	41	Alive
Female	58	3.5*3*1	T2N0M0	40	Alive
Female	72	8*5*1.5	T4N0M0	21	Dead
Male	56	5.5*4*1.5	T4N1M1	37	Dead
Male	57	3.5*2.5*0.6	T2N1M0	33	Dead
Female	80	7*5*5	T4N1M0	40	Alive
Female	64	4*3.5*4.2	T4N0M0	21	Dead
Female	62	4	T3N0M0	39	Alive

Male	64	6.5*1*0.5	T4N1M0	38	Alive
Female	39	2*2*0.5	T1N0M0	36	Alive
Male	60	4*3*1	T2N1M0	37	Alive
Male	67	6*4*3	T4N2M1	7	Dead
Male	56	6*4*2	T2N0M1	34	Dead
Male	61	5*4*3	T4N2M0	19	Dead
Male	43	9*4*1.5	T4N1M0	29	Dead
Female	80	2.3*2.2*0.5	T3NIM0	26	Dead
Male	62	2.5*2*0.7	T3N0M0	33	Alive
Male	53	5.5*4*0.5	T4N0M0	33	Alive
Male	72	3.5*3.5*0.4	T2N0M0	33	Alive
Male	57	6*6*6	T4N0M1	27	Dead
Female	67	3*3*0.4	T2N1M0	29	Dead
Male	41	5*3*0.6	T4N2M0	32	Alive
Male	87	5*4*1	T4N2M0	24	Dead
Female	58	3.5*2.5*1	T2N0M0	39	Alive
Male	50	4.5*2*1.2	T2N0M0	38	Alive
Male	77	7*6*1	T2N0M0	30	Alive
Male	69	5*3.5*1	T2N2M0	21	Alive
Male	59	3.5*3*0.5	T4N0M0	28	Dead
Male	70	5*5*1.5	T4N1M0	21	Alive
Female	79	3.5*3*1	T4aN0M0	26	Alive
Female	58	2*2*0.5	T3N0M0	23	Alive
Male	58	3*3*1	T4aN1MO	27	Alive
Male	72	2*2*0.3	T4aN2M0	22	Alive
