# Supplementary material for: Long noncoding RNA BFAL1 mediates enterotoxigenic Bacteroides fragilis-related carcinogenesis in colorectal cancer via the RHEB/mTOR pathway
Source: Cell Death Dis. 2019 Sep 12;10(9):675. doi: 10.1038/s41419-019-1925-2 (PMC6742644; doi:10.1038/s41419-019-1925-2)
Supplement: Supplementary file 5 — Supplementary Table S2 [file 41419_2019_1925_MOESM5_ESM.pdf]

Supplementary Table S2: mTOR related genes in KEGG_MTOR_SIGNALING_PATHWAY

Gene	Rank Metric Score	Running ES	Core Enrichment
VEGFB	3.161638975	0.07358044	Yes
EIF4EBP1	2.223654747	0.10691172	Yes
PGF	2.045122623	0.15341493	Yes
CAB39L	1.743314862	0.18186755	Yes
MAPK1	1.652658463	0.21956748	Yes
FIGF	1.553957105	0.25245178	Yes
ULK2	1.525366306	0.2900106	Yes
PIK3CD	1.481941104	0.3258293	Yes
AKT3	1.362404346	0.34963745	Yes
EIF4E	1.345869899	0.38330996	Yes
MLST8	1.098068476	0.37520814	Yes
RHEB	1.066649556	0.39904526	Yes
RPS6KA2	0.767386079	0.35229874	No
RPS6KB1	0.754489005	0.36953112	No
MTOR	0.708305895	0.37552634	No
RICTOR	0.487957418	0.32323012	No
STK11	0.439735293	0.31790605	No
AKT2	0.374434322	0.30450383	No
PIK3R1	0.358725429	0.30837616	No
HIF1A	0.354271799	0.31633183	No
AKT1	0.327425271	0.3135248	No
PDPK1	0.313973755	0.31725836	No
PIK3CB	0.281619281	0.31264737	No
CAB39	0.233627111	0.30192676	No
VEGFA	0.199731082	0.2947208	No
RPS6KA3	0.16075772	0.2825762	No
RPTOR	0.159043834	0.28595805	No
EIF4B	0.137072593	0.28047764	No
RPS6KA1	0.116984822	0.27503344	No
RPS6KB2	0.11019212	0.27467158	No
TSC1	-0.006499067	0.22507598	No
MAPK3	-0.039038088	0.21408999	No
RPS6	-0.104260527	0.18963517	No
TSC2	-0.105843812	0.19181104	No
DDIT4	-0.231294319	0.14886539	No
ULK3	-0.24029085	0.15128584	No
PIK3R5	-0.348122478	0.11634074	No
EIF4E2	-0.411963969	0.10248541	No
PIK3CA	-0.441056788	0.10347886	No
PIK3R2	-0.796122313	0.01224577	No
BRAF	-0.89815402	0.01054868	No
PRKAA1	-0.915527761	0.0310395	No
ULK1	-0.925451219	0.05338917	No
PIK3R3	-1.676111937	-0.0238807	No
STRADA	-1.681132317	0.02064394	No
IGF1	-1.908586502	0.05255703	No
