# Supplementary material for: Long noncoding RNA BFAL1 mediates enterotoxigenic Bacteroides fragilis-related carcinogenesis in colorectal cancer via the RHEB/mTOR pathway
Source: Cell Death Dis. 2019 Sep 12;10(9):675. doi: 10.1038/s41419-019-1925-2 (PMC6742644; doi:10.1038/s41419-019-1925-2)
Supplement: Supplementary file 6 — Supplementary Material Table [file 41419_2019_1925_MOESM6_ESM.pdf]

Supplementary Material Tables

Table 1. Primers of lncRNAs and genes

Gene	Forward primer	Reverse Primer
BFAL1	5'-TAGAGCCGAGGTCTCACTATGT-3'	5'-TCACTTTTTGGACTGGGTGT-3'
AK001058	5'-ACACCTCCGTCCAATCTCCTGAG-3'	5'-CAGTTCATGTCATGTGGCCTCTCC-3'
AK027294	5'-TTACTCAGGTGTGCTTAGGTGTGC-3'	5'-TTCCAGTCTTGGTCTGCTGTTCAC-3'
AK098156:	5'-GACGCCTGCCTCTTGTATCCAC-3'	5'-GCTGAGATCATGCCACTGTACTCC-3'
AK098081	5'-GGAGTGCAGTGGTGACATCATAGC-3'	5'-CATAGTAAGGCCGTGTGCAGTGG-3'
AK095288	5'-GCACGATCTTGGCTCACTGC-3'	5'-GCAATTAGCCGGGTGTGGTG-3'
AK123903:	5'-GCGGCCCAGCTTCTTCTTTC-3'	5'-CTAAGGTCCCGCGTCTCTGG-3'
AK098473	5'-ACCACTGTGGCTGCCCTTAA-3'	5'-TGGCCCAGTGTTTCTCCAGG-3'
RHEB	5'-GGAGGAGGAAGGTTCAGAGGAGAC-3'	5'-CTATGAGGACGGCACTCGCAATG-3'
ACTB	5'-CCTTGCACATGCCGGAG-3'	5'-RGCACAGAGCCTCGCCTT-3'
ETBF (*bft*)	5'-GGATACATCAGCTGGGTTGTAG-3'	5'-GCGAACTCGGTTTATGCAGT-3'
16S	5'-GGTGAATACGTTCCCGG-3'	5'-TACGGCTACCTTGTTACGACTT-3'
GAPDH	5'-GCATTGCCCTCAACGACCAC -3'	5'-CCACCACCCTGTTGCTGTAG -3'

Table 2. Primers of miRNA by GeneCopoeia

microRNA	Catalog
miR-155-5P	HmiRQP0221
miR-200a-3P	HmiRQP0298
miR-199a-5p	HmiRQP0290
miR-495-3p	HmiRQP053
U6	HmiRQP9001

Table 3. SiRNAs and miRNAs mimics/inhibitors

Gene Name	Forward	Reverse
BFAL1 siRNA1	5'-GGGAGGUGUUCCUAGGUAATT-3'	5'-UUACCUAGGAACACCUCCCTT-3'
BFAL1 siRNA2	5'-GAAGAGUUAUCCUUAUUCATT-3'	5'-UGAAUAAGGAUAACUCUUCTT-3'
RHEB siRNA	5'-GGUGAUCAGUUAUGAAGAATT-3'	5'-UUCUUCAUAACUGAUCACCTT-3'
Control siRNA/ mimics control	5'-UUCUCCGAACGUGUCACGUTT-3'	5'-ACGUGACACGUUCGGAGAATT-3'
miR-155-5p mimics	5'--UUAAUGCUAAUCGUGAUAGGGGU-3'	5'--CCCUAUCACGAUUAGCAUUAAUU-3'
miR-200a-3p mimics	5'--UAACACUGUCUGGUAACGAUGU-3'	5'-AUCGUUACCAGACAGUGUUAUU-3'
miR-155-5p inhibitor	5'--ACCCCUAUCACGAUUAGCAUUAA-3'	
miR-200a-3p inhibitor	5'-ACAUCGUUACCAGACAGUGUUA-3'	
Inhibitor control	5'-CAGUACUUUUGUGUAGUACAA-3'	
