# Supplementary material for: Long noncoding RNA BFAL1 mediates enterotoxigenic Bacteroides fragilis-related carcinogenesis in colorectal cancer via the RHEB/mTOR pathway
Source: Cell Death Dis. 2019 Sep 12;10(9):675. doi: 10.1038/s41419-019-1925-2 (PMC6742644; doi:10.1038/s41419-019-1925-2)
Supplement: Supplementary file 7 — WB original figures [file 41419_2019_1925_MOESM7_ESM.pdf]

WB original figures

Fig. 3F

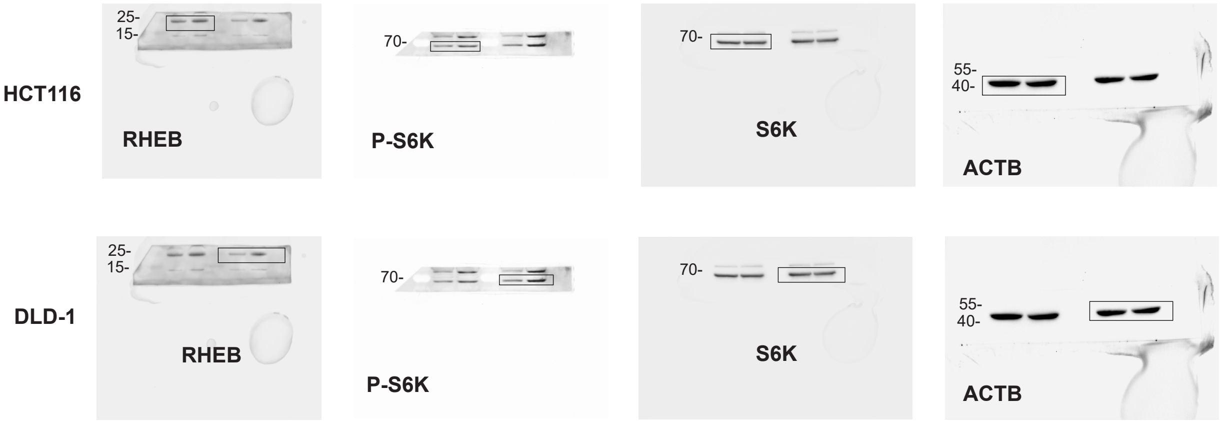

Fig. 3G

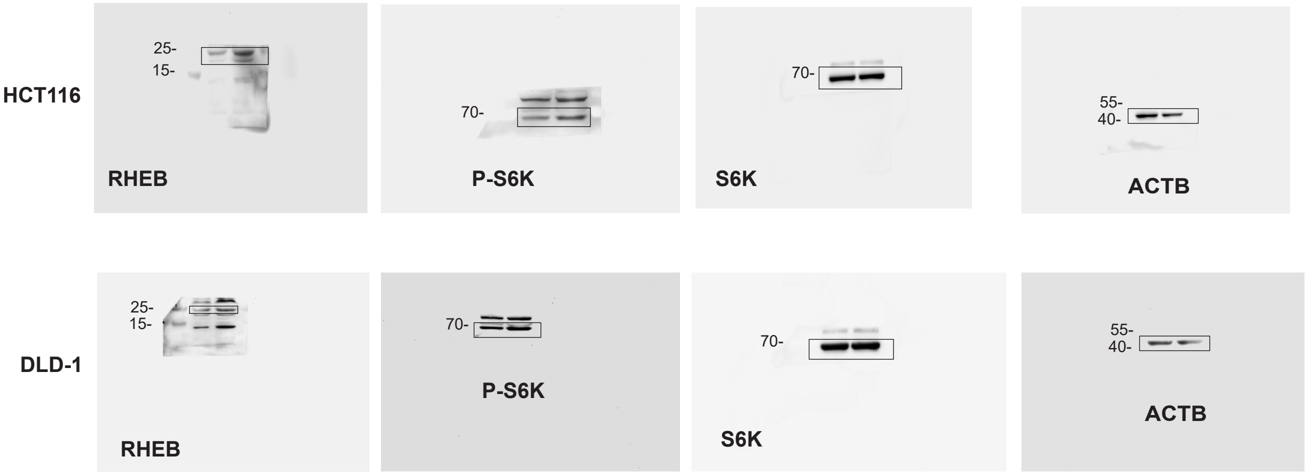

Fig. 3H

Theses WB were film- exposured in the darkroom with different exposure time.

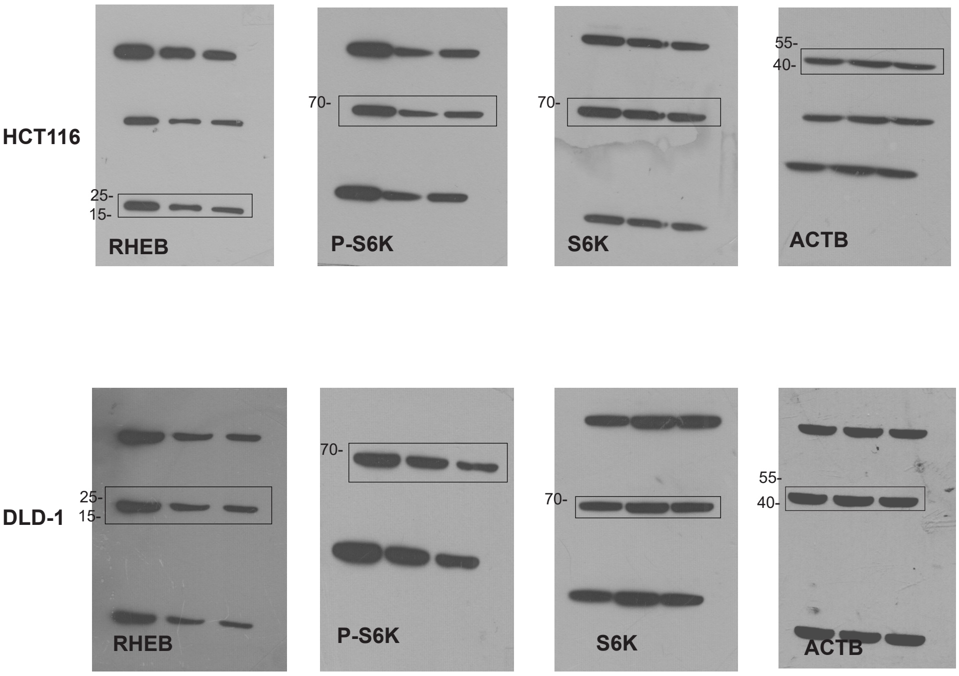

WB original figures

Fig.3I

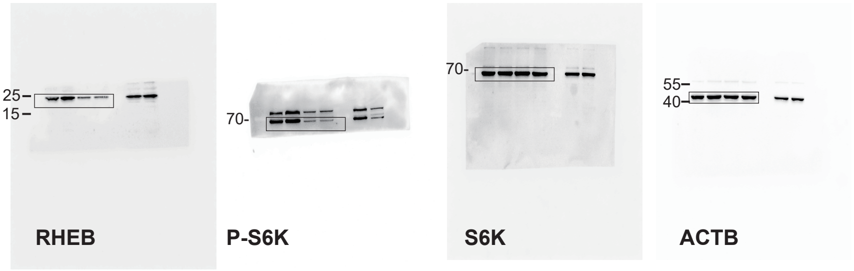

Fig. 3J

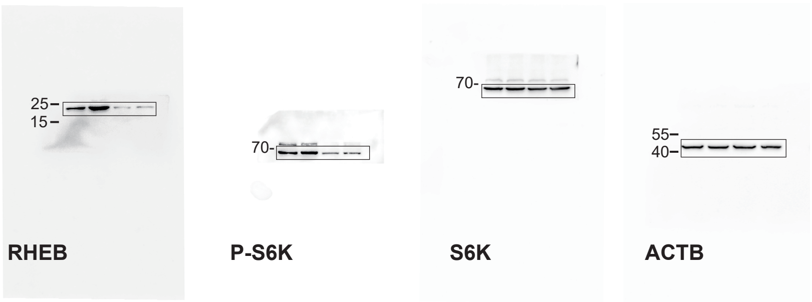

Fig. 4C

film exposure with different exposure time

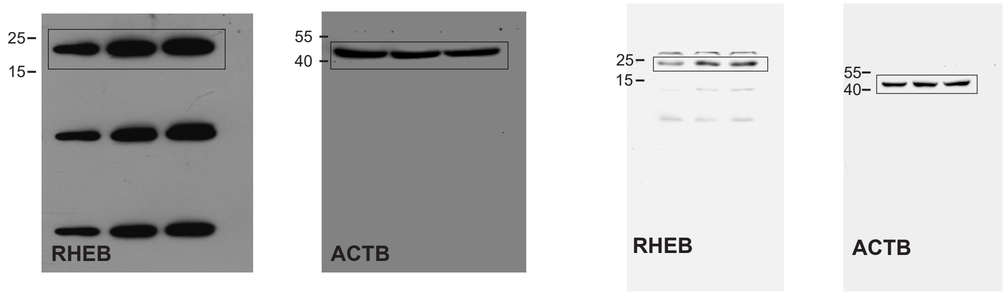

HCT116

DLD-1

Fig. 4E

film exposure with different exposure time

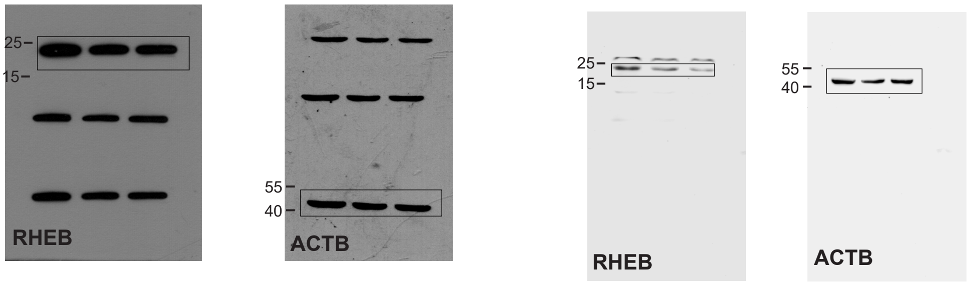

DLD-1

HCT116
