# Supplementary material for: Long noncoding RNA BFAL1 mediates enterotoxigenic Bacteroides fragilis-related carcinogenesis in colorectal cancer via the RHEB/mTOR pathway
Source: Cell Death Dis. 2019 Sep 12;10(9):675. doi: 10.1038/s41419-019-1925-2 (PMC6742644; doi:10.1038/s41419-019-1925-2)
Supplement: Supplementary file 8 — Supplementary figure legends [file 41419_2019_1925_MOESM8_ESM.docx]

**Supplementary Fig S1. The identification of lncRNA *BFAL1*, related to Figure 1**

**(A)** The screening process of *BFAL1*.

**(B)** The candidate lncRNA mRNA levels in ETBF or NTBF-treated HCT116 cells and DLD-1 cells.

**(C)** The relative mRNA expression levels of AK001058 and AK098081 in tumor tissues and pair-matched normal tissues in Renji Cohort 1.

**(D)** Comparison of AK001058 and AK098081 mRNA levels between high ETBF abundance (n = 48) and low ETBF abundance (n = 48) tissues in Renji Cohort 1.

**(E)** RACE analysis of *BFAL1*.

**(F)** The nuclear and cytoplasm fraction of *BFAL1* in HCT116 cells.

**(G)** The *ab initio* ncRNA transcriptome predictor of *BFAL1*.

**(H)** The CPC analysis of *BFAL1.*

**(I)** The relative expression of *BFAL1* in CRC cell lines and the normal colorectal epithelial cell line.

**(J)** Univariate analysis of Renji Cohort 1.

**Supplementary Fig S2. The biological function of *BFAL1*, related to Figure 2**

**(A and B)** GSEA indicating the proliferation and cell cycle biological function of *BFAL1*.

1. GO analysis in ETBF treated cells.

**(D and E)** The overexpression and knockdown transfection efficiency for *BFAL1* in HCT116 cells and DLD-1 cells.

**(F)** Representative images of Ki67-positive cells under different treatments with *BFAL1* shRNA1 (40 × magnification, Scale bars: 50 µm).

**(G)** Xenograph tumors in the nude mouse models under different treatments: Control shRNA, ETBF, *BFAL1* shRNA2, and *BFAL1* shRNA2 with ETBF (n = 6).

**(H)** Statistical analysis of tumor sizes (mean ± SD, n = 6**,** ANOVA, *******P* < 0.01).

**(I)** Tumor weight of different mice groups (mean ± SD, n = 6**,** ANOVA, *******P* < 0.01).

**(J)** Representative images of Ki67-positive cells under different treatments with *BFAL1* shRNA2 (40 × magnification, Scale bars: 50 µm).

**Supplementary Fig S3. The mechanism of *BFAL1*’s activation of the mTOR pathway, related to Figure 4 and Figure 5**

**(A)** mTOR-related gene expression in ETBF-treated DLD-1 cells.

**(B)** Luciferase assays were performed in HCT116 cells and DLD-1 cells transfected with *BFAL1* overexpression or knockdown vectors. Luciferase reporters expressing pGL3-RHEB were used.

**(C)** Representative images of Ki67-positive cells under different treatments (40 × magnification, Scale bars: 50 µm).

**Supplementary Table S1.** The clinicopathological information of the 96 CRC cases in Renji Cohort 1, including gender, age, tumor size, TNM stage, survival time, and life status.

**Supplementary Table S2.** The mTOR-related gene expression in the KEGG_MTOR_SIGNALING_ PATHWAY.

**Supplementary Material Table.** The gene primers, siRNAs, miRNAs' mimics and inhibitor sequences in this article are all listed in the tables.
